# Identification of methylation‐driven genes related to prognosis in clear‐cell renal cell carcinoma

**DOI:** 10.1002/jcp.29046

**Published:** 2019-07-05

**Authors:** Jia Wang, Qiujing Zhang, Qingqing Zhu, Chengxiang Liu, Xueli Nan, Fuxia Wang, Lihua Fang, Jie Liu, Chao Xie, Shuai Fu, Bao Song

**Affiliations:** ^1^ School of Medicine and Life Sciences University of Jinan‐Shandong Academy of Medical Sciences Jinan China; ^2^ Department of Oncology Zibo Maternal and Child Health Hospital Zibo China; ^3^ Department of Oncology, Shandong Cancer Hospital and Institute Shandong First Medical University and Shandong Academy of Medical Sciences Jinan China; ^4^ Department of Oncology Jinan Jigang Hospital Jinan China; ^5^ Department of Oncology Wu Di People Hospital Binzhou China; ^6^ Department of Oncology YunCheng Conuntry People's Hospital YunCheng China; ^7^ Department of Oncology Chang Qing District People's Hospital Jinan China; ^8^ Basic Laboratory, Shandong Cancer Hospital and Institute Shandong First Medical University and Shandong Academy of Medical Sciences Jinan China

**Keywords:** clear‐cell renal cell carcinoma, methylation‐driven genes, MethylMix, prognosis, the Cancer Genome Atlas (RRID:SCR_014514)

## Abstract

With the participation of the existing treatment methods, the prognosis of advanced clear‐cell renal cell carcinoma (ccRCC) is poor. More evidence indicates the presence of methylation in ccRCC cancer cells, but there is a lack of studies on methylation‐driven genes in ccRCC. We analyzed the open data of ccRCC in The Cancer Genome Atlas database to obtain ccRCC‐related methylation‐driven genes, and then carried out pathway enrichment, survival, and joint survival analyses. More important, we deeply explored the correlation between differential methylation sites and the expression of these driving genes. Finally, we screened 29 methylation‐driven genes via MethylMix, of which six were significantly associated with the survival of ccRCC patients. This study demonstrated that the effect of hypermethylation or hypomethylation on prognosis is different, and the level of methylation of key methylation sites is associated with gene expression. We identified methylation‐driven genes independently predicting prognosis in ccRCC, which offers theoretical support in bioinformatics for the study of methylation in ccRCC and a new perspective for the epigenetic study of ccRCC.

## Graphical abstract

In this study, a variety of algorithms, R software package, survival analysis, and combined survival analysis were used to screen the overall survival‐related methylated‐driven genes in patients with clear‐cell renal cell carcinoma (ccRCC). It was found that hypermethylation or hypomethylation had different effects on prognosis, and the level of methylation of key methylation sites was associated with gene expression. And from the biological point of view, this study provides a new perspective for the study of epigenetics of ccRCC.

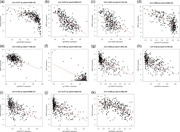

## 1 BACKGROUND

Renal cell carcinoma ranks among the top 10 in the world in the diagnosis rate of both sexes (5% for men and 2% for women; Capitanio et al., 2019), and shows an increasing trend year by year. Clear‐cell renal cell carcinoma (ccRCC) is the most common subtype (70–80%) (Greef & Eisen, 2016). About 25% of the patients have lost the opportunity of operating at the time of diagnosis (Lalani et al., 2019), and approximately only 10% of them survive 5 years later (van den Heuvel et al., 2019). And the group of patients who had an occasion of surgery had a 20% chance of postoperative recurrence (Motzer, 2016). Later, the discovery that the Von Hippel Lindau (VHL) gene is present in more than 90% of ccRCC (Nargund, Osmanbeyoglu, Cheng, & Hsieh, 2017) enriched the treatment of renal clear‐cell carcinoma. However, the response rate of the existing treatment is only about 27% recently, and at least 75% of the patients who received traditional treatment make progress 2 years later (Motzer, 2016). In spite of immunotherapy improving the overall response rate, the incidence of serious adverse events also increases (Rini et al., 2019). With the clinical efficacy of immunotherapy, the exploration of the mode of combined immunotherapy was followed, but at present, there were no reliable biomarkers for early screening and prognosis judgment of ccRCC, which limits the progress of treatment of renal clear‐cell carcinoma. Although early studies have discovered that the prognostic markers of ccRCC may be mutated genes such as VHL (Gulati et al., 2014). The heterogeneity of ccRCC itself requires more effective biomarkers to evaluate the prognosis. For this reason, there is an urgent need to ascertain valuable molecular targets in the in‐depth study of ccRCC.

Advances in research techniques have led us to a deeper understanding of the adverse diseases in our own professional field. For example, we recognize that the high expression of CD36 (cluster of differentiation 36) transcriptional group (Xu, Qu, Wang, Zhang, & Ye, 2019) and P21 (RAC1) activated kinase 1 (PAK1) protein (Qu, Liu, Bai, Xu, & Guo, 2019) is a sign of poor prognosis in ccRCC, However, BCL2 associated athanogene 1 and NOP56 ribonucleoprotein are different from CD36 transcriptional group and PAK1 protein, their high expression indicates a better prognosis (Giridhar et al., 2017). The role of gene methylation in the development of cancer has been gradually perceived and recognized. Researchers have successfully identified methylated genes or methylation‐driven genes that forecast the prognosis of esophageal squamous cell carcinoma (ESCC; Roy et al., 2019), lung squamous cell carcinoma (Gao et al., 2019), lung adenocarcinoma (C. Su et al., 2019), and hepatocellular carcinoma (G.‐X. Li et al., 2019). Moreover, the function of epigenetic abnormalities in ccRCC has been confirmed. Bioinformatics analysis of microarray data has broad prospects and clinical significance. Therefore, the era of clinical application of methylated and methylation‐driven genes is bound to come. However, there is short of previous evidence for the exploration of methylation‐driven genes in ccRCC.

The opening of a large database such as The Cancer Genome Atlas (TCGA; Tomczak, Czerwińska, & Wiznerowicz, 2015) makes it possible to meet the deeper and more accurate needs of scientists for disease exploration. It contains genetic data such as human‐methylated genes needed by the researchers, as well as a variety of clinical prognosis information, supplemented by bioinformatics technology so that we continue to have a new understanding of the occurrence and development of cancer and promote the rapid development of the discipline field. Our team collected the opened big data of TCGA and analyzed the data using the MethylMix software package (Gevaert, 2015) developed by Gevaert et al. to filter out the differentially methylated genes connected with the prognosis of ccRCC. Then, the gene enrichment pathway was visualized, and the survival and joint survival analysis were carried out to ascertain the relationship between the methylation‐driven genes and the survival of the patient. The purpose of this study was to evaluate the relationship between methylation‐driven genes, gene loci, and messenger RNA (mRNA) data for the sake of understanding the cancer mechanism involved in methylation ulteriorly and providing valuable medical evidence for the treatment and prognosis of ccRCC.

## 2 MATERIALS AND METHODS

### 2.1 Data source and data processing

We downloaded the data needed for this study from the open website of TCGA (https:/portal.gdc.cancer.gov/, RRID:SCR_014514) using the kidney renal clear‐cell carcinoma (KIRC) cohort, including methylation, mRNA expression, and clinical information in patients with ccRCC. Methylation data of 485 specimens (160 normal samples and 325 cancer samples involved) and mRNA expression data of 611 samples (comprising 72 normal specimens and 539 ccRCC specimens) were obtained.

DNA methylation data in patients with ccRCC were assayed using the Illumina Methylation 450k Bead chip (Walker et al., 2015), a technical platform for a large‐scale study of DNA methylation. And then, we exploit R‐based LIMMA (https://bioconductor.org/packages/release/bioc/html/limma.html, RRID:SCR_010943) software package (Ritchie et al., 2015) and EdgeR (https://bioconductor.org/packages/release/bioc/html/edgeR.html, RRID:SCR_012802) package (Robinson, McCarthy, & Smyth, 2010) to standardize the downloaded data to acquire differentially methylated genes (*p* = .05, logFold  Change (log FC) = 0.5) and expressed genes (FC = 5, adjusted *p* = .01). LIMMA and EdgeR are R software packages used to analyze differential genes and differentially expressed data, respectively. The operation process is as follows: the data of ccRCC were introduced into R platform, and the differentially methylated genes and differentially expressed genes were obtained and mapped after LIMMA and EdgeR packages treatment, filtration, and standardization. It is worth noting that differentially methylated genes and differentially expressed genes are genes with different degrees of methylation and different degrees of expression in normal and tumor tissues, respectively. However, the methylation‐driven genes are genes with different degree of methylation and expression between normal and tumor tissues. Therefore, the MethylMix algorithm was utilized for calculating the relationship between gene methylation level and gene expression advanced through R language with |logFC| ≥ 0, adjusted *p* < .05, and correlation coefficient (Cor) < −0.3 as screening conditions. MethylMix (https://bioconductor.riken.jp/packages/3.1/bioc/html/MethylMix.html) is an algorithm based on the β‐mixed model to identify methylation states and compares them with the normal DNA methylation state. It implemented the differential methylation value, that is, the difference between the tumor methylation state and the normal methylation state, to identify disease‐related methylation‐driven genes. And the operation of MethylMix package requires the input of three specific data sets (the methylation data of the tumor samples, the methylation data of the normal tissue samples, and the corresponding gene expression data of the tumor samples). As a result, we submit the data to MethylMix according to the requirements of the algorithm, and finally screened out differentially methylation‐driven genes for survival analysis. All the data we download is open to the TCGA platform, so we do not need the approval of the local ethics committee.

### 2.2 Path enrichment analysis of methylation‐driven genes

To further comprehend the biological functions of these methylation‐driven genes, we applied ConensusPathDB (http://cpdb.molgen.mpg.de/; Herwig, Hardt, Lienhard, & Kamburov, 2016) to visualize the analysis of pathway of methylation‐driven genes, and took the cut‐off value of *p* value = .05 as the criterion. ConensusPathDB integrates the interaction networks of *Homo sapiens*, containing signal transduction, gene regulation, and drug‐target interaction, and biochemical metabolism either, which currently integrates 32 public databases. It can interact with the genetic information we collected to avoid redundancy perfectly, so it has become one of the favorite tools for visual methylation‐driven gene pathway enrichment analysis in the research process.

### 2.3 Survival analysis and joint survival analysis

To explore the effect and significance of methylation‐driven genes on the prognosis of patients with ccRCC, we take the overall survival (OS) of patients, that is, from the time when the patient is confirmed to have the disease and to the time of death (for any reason), as the survival time and use it for final prognosis evaluation, survival analysis, and joint survival analysis. Survival analysis was used to show the relationship between the hypermethylated/hypomethylated level of genes and overall survival, while combined survival analysis was used to analyze the effect of methylated level combined with gene expression on survival. Therefore, we used Kaplan‐Meier curve (Schultz, Peterson, & Breslau, 2002) to analyze and evaluate the relationship between methylation‐driven genes and survival rate, and verified this correlation by Log‐rank test (Koletsi & Pandis, 2017) on the survival R software package platform (Singh & Mukhopadhyay, 2011), so as to screen the possibility of independent prognosis of methylation‐driven genes. And the setting of *p* < .05 was viewed as statistically significant. Then, the survival R package was further used to obtain the joint survival curve in the joint survival analysis to analyze the relationship between gene methylation level and expression and prognosis of ccRCC. It is worth noting that, based on the downloaded clinical prognostic information and the information of the related sites of the methylation‐driven genes, we combined the prognostic key genes obtained from the survival analysis and the combined analysis to make sure the correlation between the expression of methylation‐driven genes and the methylated sites of key genes through the Kaplan‐Meier curve drawn by the survival R package (set *p* < .05, |Cor| > 0.5 as the screening condition).

## 3 RESULTS

### 3.1 Acquisition of methylation‐driven genes

**Figure 1:**
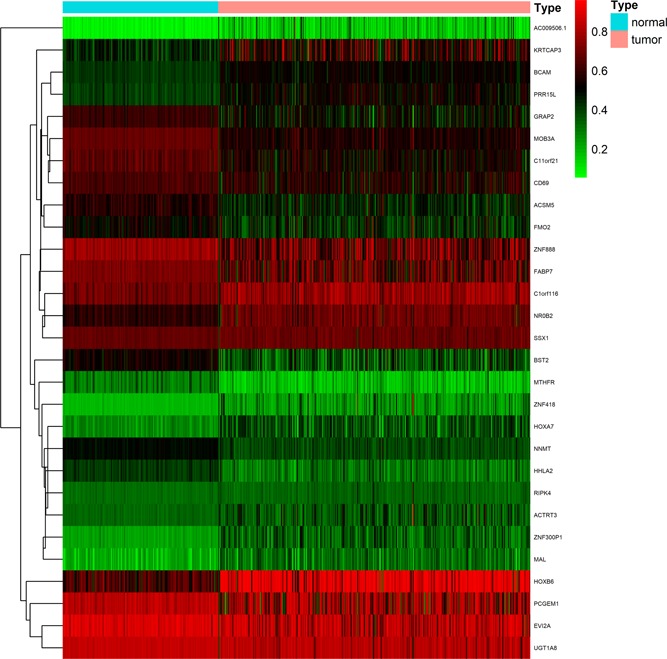
Thermal map of methylation‐driven genes associated with ccRCC. (The color change from green to red in the heat map illustrates the trend from low to high methylation. |log FC| ≥ 0, adjusted *p* < .05 and Cor < −0.3). ccRCC, clear‐cell renal cell carcinoma; FC, fold change [Color figure can be viewed at wileyonlinelibrary.com]

**Figure 2:**
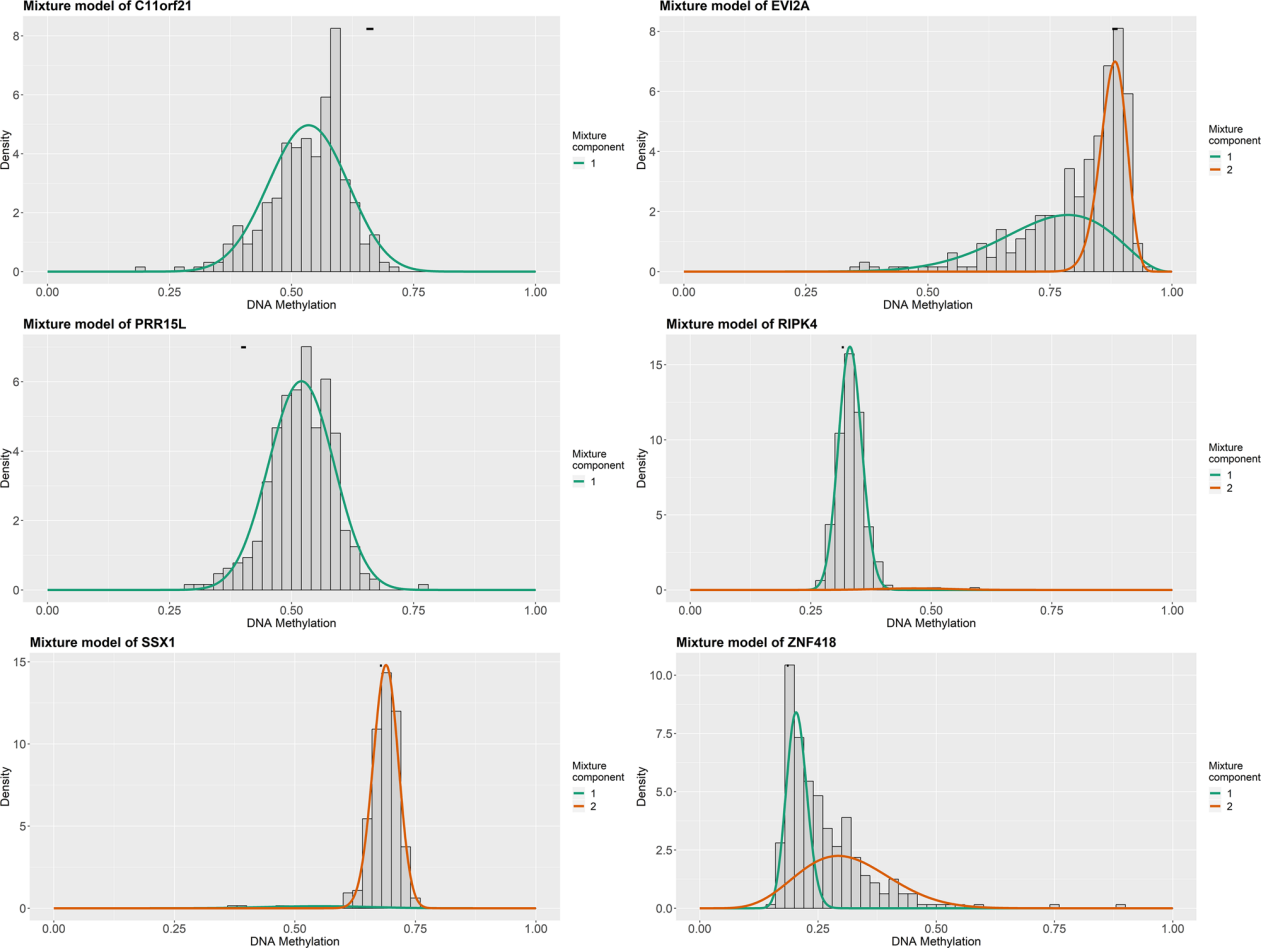
Methyl mixed Model of C11orf21, EVI2A, PRR15L, RIPK4, SSX1, and ZNF418. The distribution map represents the methylated status of methylated genes. The histogram demonstrates the distribution of methylation in tumor samples. Horizontal black bars show the distribution of methylation in normal samples. C11orf21, chromosome 11 open reading frame 21; EVI2A, ecotropic viral integration site 2A; PRR15L, proline‐rich protein 15; RIPK4, receptor‐interacting serine/threonine kinase protein 4; SSX1, SSX family member 1; ZNF418, zinc finger protein 418 [Color figure can be viewed at wileyonlinelibrary.com]

**Figure 3:**
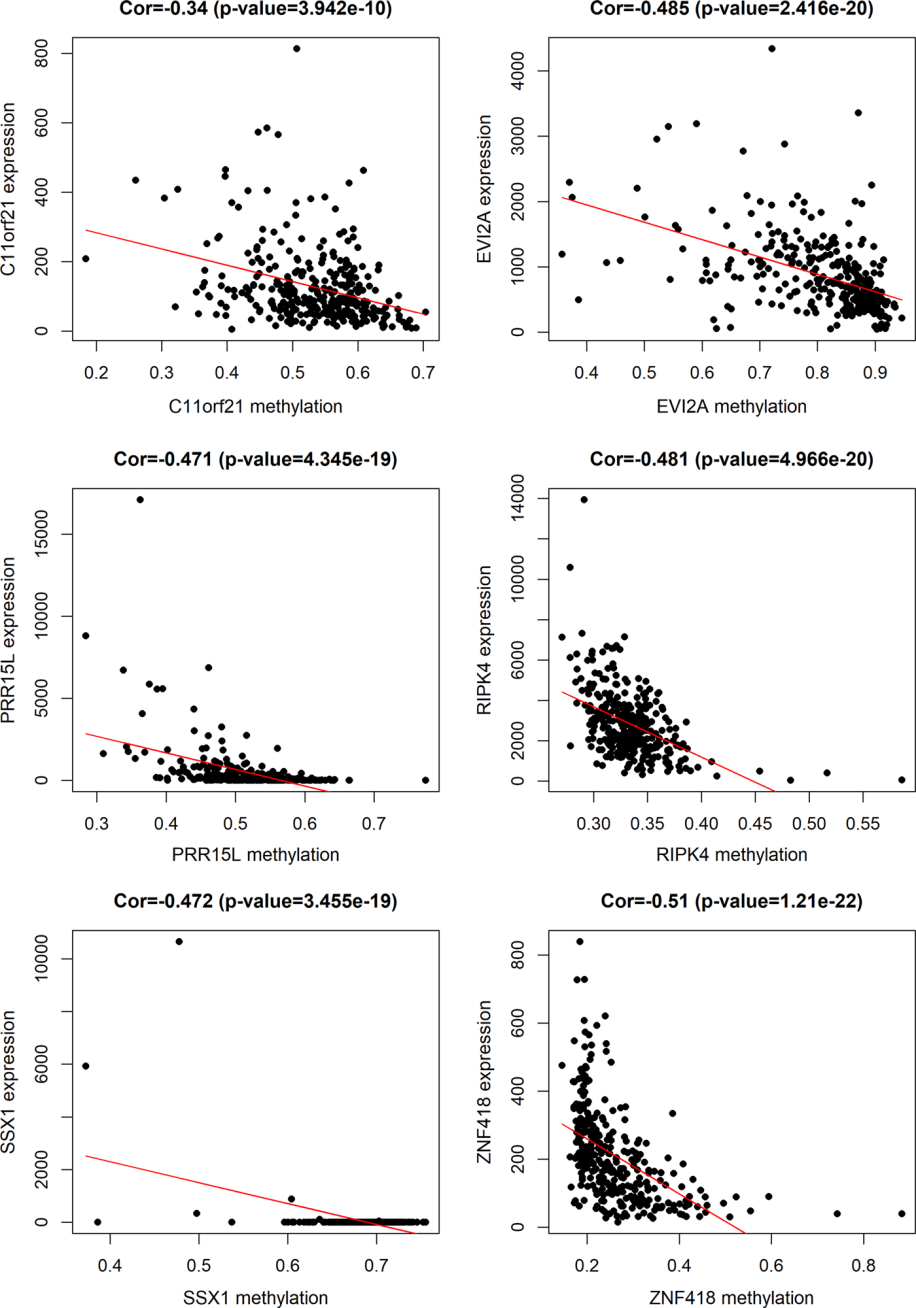
The correlation between methylation and gene expression of C11orf21, EVI2A, PRR15L, RIPK4, SSX1, and ZNF418. C11orf21, chromosome 11 open reading frame 21; EVI2A, ecotropic viral integration site 2A; PRR15L, proline‐rich protein 15; RIPK4, receptor‐interacting serine/threonine kinase protein 4; SSX1, SSX family member 1; ZNF418, zinc finger protein 418 [Color figure can be viewed at wileyonlinelibrary.com]

To study the methylation‐driven genes associated in patients with ccRCC, we downloaded methylated data from 160 normal specimens and 325 methylated cancer samples, as well as mRNA expression data from 72 normal specimens and 539 ccRCC samples from the TCGA database. First of all, we used LIMMA software package and EdgeR package to extract abnormal methylation data and gene expression data, and obtained 105 differentially methylated genes and 257 differentially expressed genes, respectively. Second, the data were integrated and grouped, and 29 methylation‐driven genes were screened via using MethylMix R package (Table 1). Afterward the connection between gene methylation and gene expression was visualized on the R platform (Figure 1). Six of the most representative of these genes (chromosome 11 open reading frame 21 [C11orf21], ecotropic viral integration site 2A [EVI2A], proline‐rich protein 15 [PRR15L], receptor‐interacting serine/threonine kinase protein 4 [RIPK4], SSX family member 1 [SSX1], and zinc finger protein 418 [ZNF418]) are shown in Figures 2 and 3 and the rest in Figures S1 and S2.

**Table 1:** Twenty‐nine methylation‐driven genes in ccRCC

Gene	normal Mean	Tumor mean	log FC	adjust *P*	cor
NNMT	0.490801045	0.386640567	−0.34415	1.78E−64	−0.319852
MOB3A	0.683159551	0.557690028	−0.29276	2.89E−61	−0.429547
BCAM	0.412729523	0.515318674	0.320268	1.51E−58	−0.478336
PRR15L	0.401467303	0.519562987	0.372016	4.53E−54	−0.470521
C11orf21	0.660522453	0.533692924	−0.3076	1.86E−53	−0.340058
HOXB6	0.624464597	0.866363149	0.472352	7.77E−53	−0.57175
ZNF300P1	0.226865565	0.341288606	0.589155	1.43E−52	−0.436561
HHLA2	0.410567575	0.304387165	−0.43171	8.02E−52	−0.494496
BST2	0.535399458	0.37064583	−0.53057	4.00E−51	−0.426027
NR0B2	0.578674355	0.686782026	0.247101	2.12E−50	−0.431367
MTHFR	0.298172025	0.182097665	−0.71143	7.85E−50	−0.3702
ACSM5	0.570660517	0.451334871	−0.33843	2.05E−48	−0.477774
AC009506.1	0.082717108	0.167930097	1.021603	1.13E−46	−0.399622
MAL	0.201505706	0.313709011	0.638606	3.03E−45	−0.345857
GRAP2	0.601094479	0.489241065	−0.29705	1.16E−43	−0.342031
ZNF418	0.185958338	0.260853528	0.488261	2.18E−40	−0.509882
HOXA7	0.266592109	0.364415225	0.450949	6.13E−33	−0.41498
C1orf116	0.699315024	0.761773712	0.12342	5.73E−27	−0.530969
KRTCAP3	0.443374147	0.578722251	0.384346	4.80E−26	−0.577152
CD69	0.629105907	0.553558638	−0.18457	2.19E−25	−0.364012
FMO2	0.516856494	0.438365926	−0.23763	5.60E−22	−0.48796
EVI2A	0.883065324	0.809783928	−0.12498	5.21E−18	−0.485002
ZNF888	0.776904328	0.67156532	−0.21021	5.46E−14	−0.408986
RIPK4	0.316498368	0.334545342	0.080004	2.16E−13	−0.481455
UGT1A8	0.846779043	0.813971416	−0.05701	4.47E−13	−0.413703
PCGEM1	0.827941383	0.72499483	−0.19156	3.29E−07	−0.330849
ACTRT3	0.328218126	0.369746258	0.171881	1.57E−06	−0.431851
FABP7	0.723408963	0.643630606	−0.16858	1.29E−05	−0.624072
SSX1	0.678468784	0.683130154	0.009878	0.0004706	−0.471696

*Note*: ACSM5, acyl‐CoA synthetase medium chain family member 5; ACTRT3, actin‐related protein T3; BCAM, basal cell adhesion molecule; BST2, bone marrow stromal cell antigen 2; C11orf21, chromosome 11 open reading frame 21; C1orf116, chromosome 1 open reading frame 11; ccRCC, clear‐cell renal cell carcinoma; CD69, cluster of differentiation 69; EVI2A, ecotropic viral integration site 2A; FABP7, fatty acid binding protein 7; FMO2, flavin containing monooxygenase 2; GRAP2, GRB2 related adaptor protein 2; HHLA2, HERV‐H LTR‐associating protein 2; HOXA7, homeobox A7; HOXB6, homeobox B6; KRTCAP3, keratinocyte associated protein 3; MAL, Mal T cell differentiation protein; MOB3A, MOB kinase activator 3A; MTHFR, methylenetetrahydrofolate reductase; NNMT, nicotinamide N‐methyltransferase; NR0B2, nuclear receptor subfamily 0 Group B member 2; PCGEM1, PCGEM1 prostate‐specific transcript; PRR15L, proline‐rich protein 15; RIPK4, receptor‐interacting serine/threonine kinase protein 4; SSX1, SSX family member 1; UGT1A8, UDP‐glucuronosyltransferase 1‐8; ZNF300P1, zinc finger protein 300 pseudogene 1; ZNF418, zinc finger protein 418; ZNF888, zinc finger protein 888.

### 3.2 Path enrichment analysis of methylation‐driven genes

**Figure 4:**
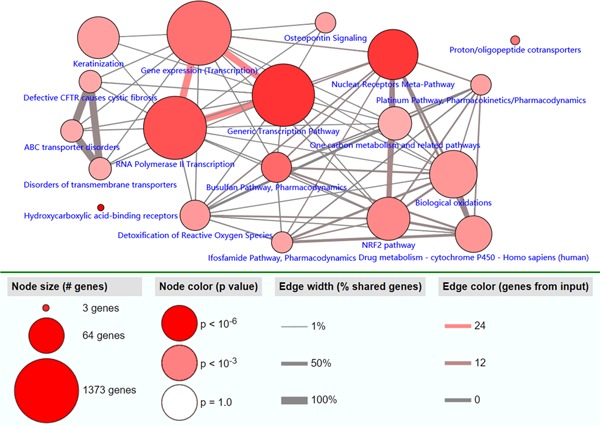
Pathway enrichment analysis of ccRCC‐related aberrant methylation‐driven genes by using ConsensuspathDB (only the pathways in which *p* < .01 are shown here). Node size: the number of genes; node color: *p* value; edge width: percentage of shared genes; edge color: genes from input; ccRCC, clear‐cell renal cell carcinoma [Color figure can be viewed at wileyonlinelibrary.com]

We utilized ConensusPathDB online software for path enrichment analysis to further study the mechanism of methylation‐driven genes in the occurrence and development of ccRCC. The results of pathway enrichment analysis showed that the methylation‐driven genes were mainly enriched in the universal transcriptional pathway, RNA polymerase II transcription and gene expression, and were significantly related to the enriched events (Figure 4).

### 3.3 Prognostic evaluation and survival analysis

**Figure 5:**
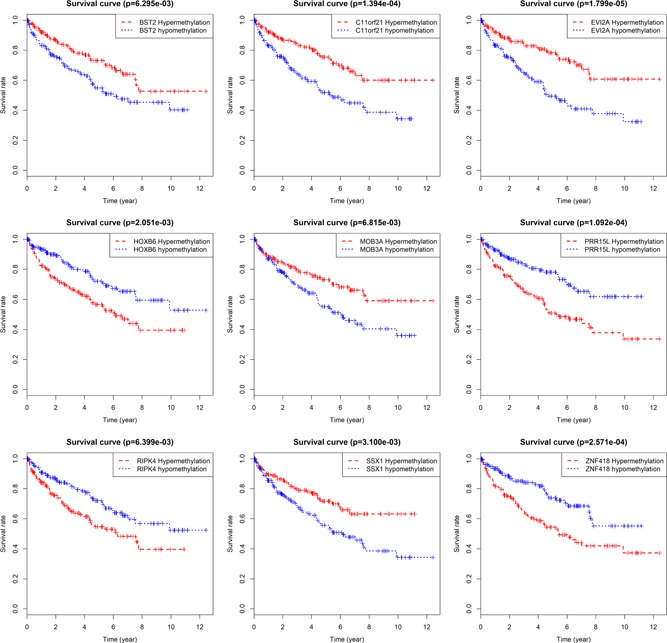
Kaplan‐Meier survival curves of nine driving genes. BST2, bone marrow stromal cell antigen 2; C11orf21, chromosome 11 open reading frame 21; EVI2A, ecotropic viral integration site 2A; HOXB6, homeobox B6; MOB3A, MOB kinase activator 3A; PRR15L, proline‐rich protein 15; RIPK4, receptor‐interacting serine/threonine kinase protein 4; SSX1, SSX family member 1; ZNF418, zinc finger protein 418 [Color figure can be viewed at wileyonlinelibrary.com]

The prognostic value of 29 methylation‐driven genes was construed by survival R software package, and nine genes were detected to be independent prognostic indicators of ccRCC (Figure 5). Among them, the methylation levels of EVI2A, C11orf21, SSX1, bone marrow stromal cell antigen 2 (BST2), and MOB kinase activator 3A (MOB3A) were significantly positively correlated with the OS of patients with ccRCC, while the other four genes (PRR15L, ZNF418, homeobox B6 [HOXB6], RIPK4) were inversely connected with survival.

**Figure 6:**
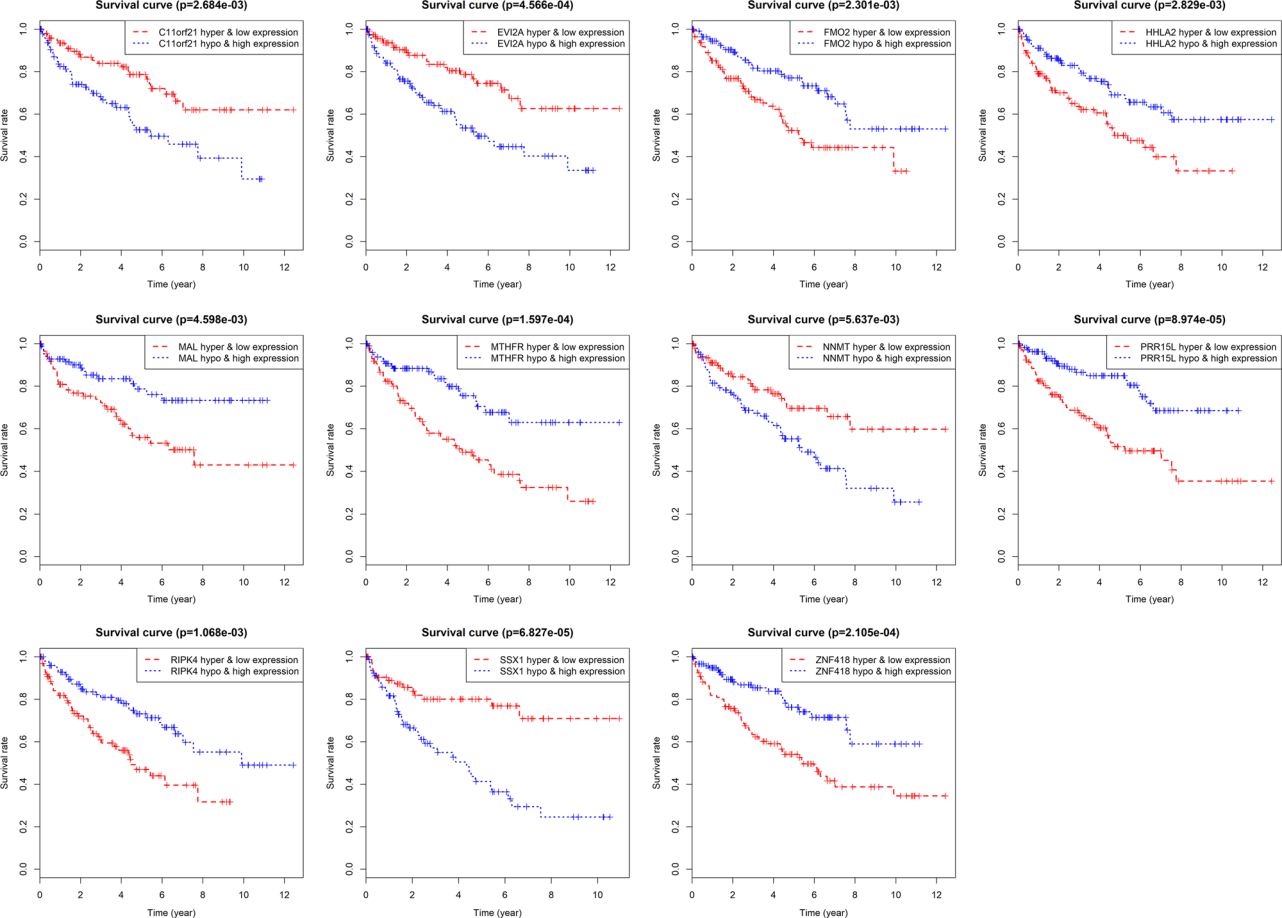
Results of combined survival analysis of 11 genes. Regard *p* < .05 as statistically significant, C11orf21, chromosome 11 open reading frame 21; EVI2A, ecotropic viral integration site 2A; FMO2, flavin containing monooxygenase 2; HHLA2, HERV–H LTR‐associating protein 2; MAL, Mal T cell differentiation protein; MTHFR, methylenetetrahydrofolate reductase; NNMT, nicotinamide *N*‐methyltransferase; PRR15L, proline‐rich protein 15; RIPK4, receptor‐interacting serine/threonine kinase protein 4; SSX1, SSX family member 1; ZNF418, zinc finger protein 418 [Color figure can be viewed at wileyonlinelibrary.com]

Besides, for the sake of studying the correlation between methylation‐driven genes, gene expression and the prognosis of patients, we coalesced these data for analysis, with *p* < .05 as statistically significant; and found that the gene expression and methylation levels of 11 genes (SSX1, PRR15L, methylenetetrahydrofolate reductase [MTHFR], ZNF418, EVI2A, RIPK4, flavin containing monooxygenase 2 [FMO2], C11orf21, HERV‐H LTR‐associating protein 2 [HHLA2], Mal T cell differentiation protein [MAL], nicotinamide *N*‐methyltransferase [NNMT]) were significantly correlated with the prognosis of patients (Figure 6). The hypermethylation/low expression levels of SSX1, EVI2A, C11orf21, and NNMT were significantly higher than those of the patients with hypomethylation/high expression levels. On the contrary, the high methylated/low expression of the other seven genes represented poor survival.

**Figure 7:**
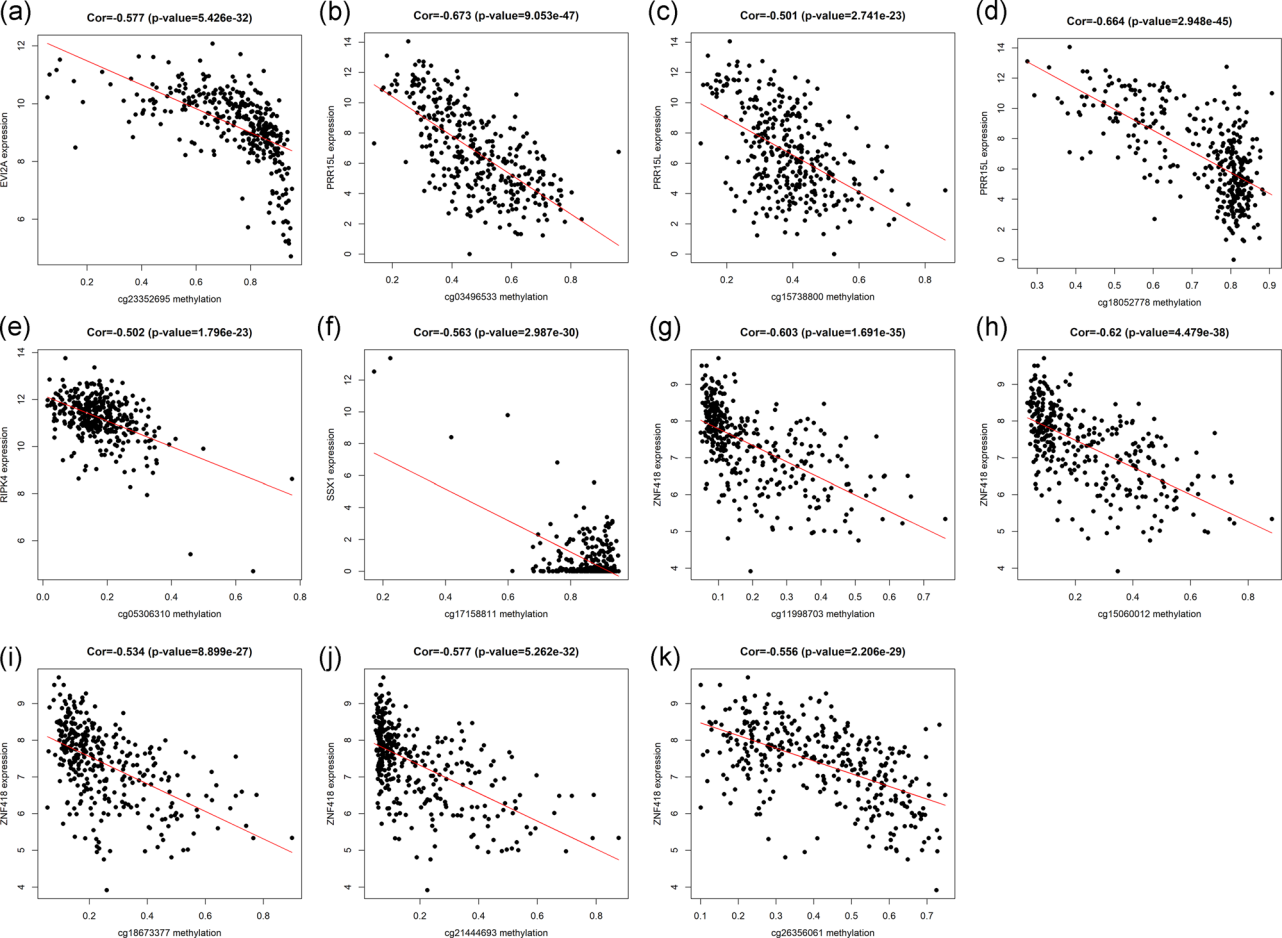
The correlation between methylated sites and matched driving gene expression of EVI2A (a), PRR15L (b–d), RIPK4 (e), SSX1 (f) and ZNF418 (g–k). (*p* < .05, |Cor| > 0.5), EVI2A, ecotropic viral integration site 2A; PRR15L, proline‐rich protein 15; RIPK4, receptor‐interacting serine/threonine kinase protein 4; SSX1, SSX family member 1; ZNF418, zinc finger protein 418 [Color figure can be viewed at wileyonlinelibrary.com]

On the basis of the above, by taking the intersection of survival analysis and joint survival analysis, we locked six prognostic genes C11orf21, EVI2A, PRR15L, RIPK4, SSX1, and ZNF418 as independent prognostic factors or biomarkers, and explored the correlation between the expression of each gene and the corresponding methylated sites, our team found that not all methylation sites were associated with the expression of driving genes. We gained sites significantly related to C11orf21 and EVI2A, PRR15L, RIPK4, SSX1, and ZNF418, respectively. The details are as follows: zero site in C11orf21, one site in EVI2A (cg23352695), three sites in PRR15L (cg03496533, cg18052778, cg15738800), one site in RIPK4 (cg05306310), one site in SSX1 (cg17158811), and five sites in ZNF418 (cg15060012, cg11998703, cg21444693, cg26356061, cg18673377; Table 2). We ascertained that there was no significant correlation between the expression of C11orf21 methylation‐driven gene and the level of methylation at all its corresponding sites (Figure S3), while the other five genes were negatively related to the level of methylation at the corresponding sites (Figure 7).

**Table 2:** Correlation between methylation sites and gene expression

Gene symbol	Methylation site	Correlation	*p* Value
EVI2A	cg23352695	−0.577	5.43E−32
PRR15L	cg03496533	−0.673	9.05E−47
	cg18052778	−0.664	2.95E−45
	cg15738800	−0.501	2.74E−23
RIPK4	cg05306310	−0.502	1.8E−23
SSX1	cg17158811	−0.563	2.99E−30
ZNF418	cg15060012	−0.62	4.48E−38
	cg11998703	−0.603	1.69E−35
	cg21444693	−0.577	5.26E−32
	cg26356061	−0.556	2.21E−29
	cg18673377	−0.534	8.9E−27

*Note*: EVI2A, ecotropic viral integration site 2A, PRR15L, proline‐rich protein 15; RIPK4, receptor‐interacting serine/threonine kinase protein 4; SSX1, SSX family member 1, ZNF418, zinc finger protein 418.

## 4 DISCUSSION

Nearly, 80% to 90% of renal cell carcinoma is clear‐cell carcinoma of the kidney (Ljungberg et al., 2015), which is one of the deadliest kidney masses in the world, with an estimated 140,000 deaths from ccRCC each year (Y. Huang et al., 2019). There is growing evidence that intratumoral heterogeneity, prevalent in ccRCCs, may affect therapeutic effect to a large extent (Crusz et al., 2016). Studies have shown that the changes in gene expression caused by methylation are connected with the development and regulation of a variety of human malignant tumors. Otherwise, unlike genetic changes, the epigenetic changes reason from methylation can be reversed (Zhao et al., 2018). Hence, it has gradually attracted extensive attention of researchers because it is noninvasive for diagnosis and detection of cancer (Luttmer et al., 2016); but the early screening of ccRCC markers are lacking recently, coupled with the limited accuracy, so the new prognostic biomarkers are in sole need of further exploration. High‐throughput sequencing technology gives us a higher ability to detect promising biomarkers correlated with the prognosis and treatment of ccRCC. In recent years, the view that epigenetic changes such as methylation are participated in the progression of ccRCC has been confirmed (Shenoy et al., 2015), and the relationship between gene methylation and ccRCC has been explored more and more. It has been found that the methylation of promoter regions of ion transport mechanism is affiliated with the risk of ccRCC (Deckers et al., 2017), respectively. The high/low methylation of DNA is associated with the invasiveness of the disease and can significantly affect the survival of patients (X. Su et al., 2017). Moreover, van Vlodrop et al. (2017) demonstrate that the four gene promoters composed of Gremlin 1, DAN Family BMP Antagonist, neuralized E3 ubiquitin protein ligase 1, Ladinin 1, and neurofilament heavy can be used as prognostic markers of ccRCC. However, there is no report on the study of abnormal methylation driving genes in ccRCC. And so far, only one methylation‐driven genes, Cytohesin 1 Interacting protein, has been reported to have an effect on ccRCC in previous studies, and the paper concludes that hypermethylation of this driver gene may be a feature of good prognosis in KIRC (Gevaert, Tibshirani, & Plevritis, 2015). Thus, it is pressed for searching new prognosis‐related driving genes in ccRCC for more accurate individualized treatment and good prognosis evaluation.

In this study, we detected TCGA data to explore the relationship between methylation‐driven genes and the prognosis of ccRCC to determine effective prognostic biomarkers. First, Limma and EdgeR packages were carried out to process the data, and then 29 methylation‐driven genes were screened based on MethylMix algorithm. Then, we made use of pathway analysis to further understand their biological functions. The results revealed that methylated genes were mainly attached oneself to universal transcriptional pathway, RNA polymerase II transcription and gene expression (transcription). It was a hint that the interaction and possible synergistic effect of methylation‐driven genes on the gene function level of renal clear‐cell carcinoma cell proliferation. Then, nine genes (EVI2A, C11orf21, SSX1, BST2, MOB3A, PRR15L, ZNF418, HOXB6, and RIPK4) were won as independent prognostic indicators by using survival R software package. In addition, 11 genes (SSX1, PRR15L, MTHFR, ZNF418, EVI2A, RIPK4, FMO2, C11orf21, HHLA2, MAL, and NNMT) were discovered to be significantly correlated with the prognosis of patients (*p* < .05) in joined survival analysis so as to ulteriorly study the relationship between the expression of methylation‐driven genes and the prognosis of patients. In conclusion, we speculated that hypermethylation of methylation‐driven genes affects the metabolic level of cells by inhibiting the expression of genes, which resulted in a decline in the survival of patients with ccRCC. Through the intersection of survival analysis and joint survival analysis, we identified six specific methylation‐driven genes (PRR15L, C11orf21, ZNF418, EVI2A, RIPK4, and SSX1) to explore the relationship between their methylation levels and methylation sites.

Among the six genes, the high methylated level of PRR15L, ZNF418, and RIPK4 prognosticated low survival rate, and the form of high methylated/low expression represented poor prognosis. PRR15L is a protein‐coding gene located on chromosome 17 and expressed in renal tissue. According to statistics, there are deficient studies on PRR15L gene at present, and this gene is reported for the first time as an index to evaluate the prognosis of ccRCC by us. ZNF418 gene encodes a zinc finger protein, which plays a negative regulatory role in mitogen‐activated protein kinase signaling pathway (Y. Li et al., 2008). Its low expression is concerned with the occurrence and development and poor prognosis of gastric cancer (Hui et al., 2018), and can be used as a biomarker for the diagnosis of ESCC (Pu et al., 2017). The overexpression of RIPK4 in receptor interaction is involved in the growth of some tumor cells (X. Huang et al., 2013). And there are some studies that showed that RIPK4 can facilitate the differentiation of epidermis through phosphorylation and participate in the carcinogenesis of skin (Lee et al., 2017) and encouraged the occurrence of nasopharyngeal carcinoma (Gong, Luo, Yang, Jiang, & Liu, 2018). Furthermore, protein kinase RIPK4 promotes the invasiveness of bladder urothelial carcinoma (Liu et al., 2018) and pancreatic cancer (Qi et al., 2018) through nuclear factor‐κB pathway and RAF1/MEK/ERK pathway, respectively. Equally important, it is closely correlated with bone metastasis of breast cancer (Zhang, He, & Zhang, 2019) and lymph node metastasis of cervical cancer (Azizmohammadi et al., 2017). We made the conclusion that patients with high methylation level of PRR15L, ZNF418, and RIPK4 had a poor prognosis compared with patients with low methylation level of methylation cleared. Moreover, combined survival analysis further displayed that their high methylated/low expression was a feature of low survival rate.

The lower the methylation level of the other three genes EVI2A, C11orf21, and SSX1, the worse the survival, and the combined survival analysis uncovered that the patients with low methylated/high expression had worse prognosis than those with high methylated/low expression. EVI2A is a protein‐coding gene that can be employed to diagnose diseases (Lo, Shen, Baumgarner, Cramer, & Lossie, 2012). In addition, some studies have revealed that it is also a specific tumor suppressor factor of lymphocytes (X.‐W. Li et al., 2014), and the upregulation of EVI2A gene expression may increase the malignant risk of malignant peripheral schwannoma (MPNST; Pasmant et al., 2011). In our study, patients with hypomethylation/high expression of EVI2A gene had a low survival rate, which was consistent with the study of MPNST. C11orf21 located on chromosome 11p15.5, in the meta‐analysis of chronic lymphocytic leukemia (CLL), S. I. Berndt et al found that C11orf21 is related to the arises of CLL (Berndt et al., 2013), but it needs to be further verified. The expression of SSX1 gene is related to the poor prognosis of patients with colon cancer (Hilal, Novikov, Novikov, & Karaulov, 2017). Moreover, Panigrahi et al discovered that specific chromosome translocation t (X; 18; p11; q11) is a feature of synovial sarcomas (SS), so SS18‐SSX1 fusion gene can be applied to the diagnose of SS (Panigrahi et al., 2018). In our study, we demonstrated that EVI2A, C11orf21, and SSX1 are effective prognostic markers of ccRCC, and their hypomethylation leads to poor prognosis. Furthermore, combined survival analysis further corroborated that the hypomethylation/high expression of the gene predicted low survival rate. Finally, it was found that the related sites of EVI2A (cg23352695), PRR15L (cg03496533, cg18052778, cg15738800), RIPK4 (cg05306310), SSX1 (cg17158811) and ZNF418 (cg15060012, cg11998703, cg21444693, cg26356061, cg18673377) driving genes were negatively correlated with their expression levels, which may result from the downregulation of ccRCC gene expression that caused by hypermethylation. Otherwise, none of the methylation sites had a statistically positive or negative relationship with the C11orf21 driving gene, which may be due to the limitations of our research. Therefore, further experiments are needed to verify the related sites found in this study and to find the sites that are not clear in this study. And we believe that the study of methylation sites of driving genes will play an important role in our further study of the mechanism.

In a word, the development of bioinformatics provides more and more evidence for the important role of DNA methylation in tumor. The six effective genes obtained in this study have been studied more or less except PRR15L, whereas, their research in ccRCC is still in the blank period. Our study discussed the role of PRR15L, C11orf21, ZNF418, EVI2A, RIPK4, and SSX1 in ccRCC for the first time. We used R software to analyze the methylation‐driven genes of ccRCC in TCGA data. After that, for the sake of verifying the relationship between the degree of gene methylation and the prognosis of patients, we utilized survival analysis. In addition, to further ensure the effect of its practical application, we united the level of gene methylation, gene expression and patient prognosis survival information for joint survival analysis, and finally carried out a more accurate site analysis. This study has found potential methylation‐driven genes that can be used as biomarkers of ccRCC, and the exploration of related sites has further refined our research, giving us a new understanding of ccRCC from the perspective of bioinformatics. However, it is undeniable that these ccRCC‐related differential driving genes need further experimental verification on the basis of a rigorous attitude.

## 5 CONCLUSION

To sum up, we conducted R soft packages and MethylMix technology to analyze the data we got from TCGA, and the outcomes was 29 methylation‐driven genes which have a close relevance with ccRCC. Afterwards, we explored the connection of driving gene methylation, gene expression and the prognosis of patients via survival analysis and joint survival analysis on the basis of available data, and identified the methylation‐driven genes, EVI2A, C11orf21, SSX1, PRR15L, ZNF418, and RIPK4, which can be used as prognostic markers of ccRCC. It is interesting to note that the expression levels of the five methylation‐driven genes except C11orf21 are negatively correlated with the degree of methylation of the sites. Therefore, the development and evolution of ccRCC may be due in part to the methylation of these driving genes, and the genes we obtained in this study can be used as prognostic markers for ccRCC. In conclusion, the results of our study provide new ideas for the diagnosis, therapy, and prognosis of ccRCC, so that the methylation‐driven genes contribute to ccRCC discovered, and open the way for the future phase of methylation‐driven genes into clinical application.

## CONFLICT OF INTERESTS

The authors declare that there are no conflict of interests.

## AUTHOR CONTRIBUTIONS

J.W. and Q.J.Z. contributed to the design of study, J.W., Q.Q.Z. and C.X.L. performed data analysis, X.L.N. and F.X.W. collected research data, L.H.F. and J.L. provided analytical tools; Q.J.Z. and B.S. involved in drafting the manuscript, C.X. and S.F. revising the manuscript critically. All the authors were fully involved in the writing of the paper and gave final approval to the version we submitted.

## DATA ACCESSIBILITY

The data that support the findings of this study are openly available in TCGA at https:/portal.gdc.cancer.gov/ (RRID:SCR_014514)

## Supporting information


**Supporting Information Figure S1: docx.** Methyl mixed Model of other methylation‐driven genes. The distribution map represents the methylated status of methylated genes. The histogram demonstrates the distribution of methylation in tumor samples. Horizontal black bars show the distribution of methylation in normal samples

**Supporting Information Figure S2: tiff.** the correlation between methylation and Gene expression of other genes

**Supporting Information Figure S3:tiff.** Relationship between the expression of C11orf21 gene and the methylation level of all its corresponding sites (There are no methylation sites significantly associated with gene expression), C11orf21: Chromosome 11 Open Reading Frame 21
