# Supplementary figures and images for: Identification of methylation‐driven genes related to prognosis in clear‐cell renal cell carcinoma
Source: J Cell Physiol. 2019 Jul 5;235(2):1296–308. doi: 10.1002/jcp.29046 (PMC6899764; doi:10.1002/jcp.29046)

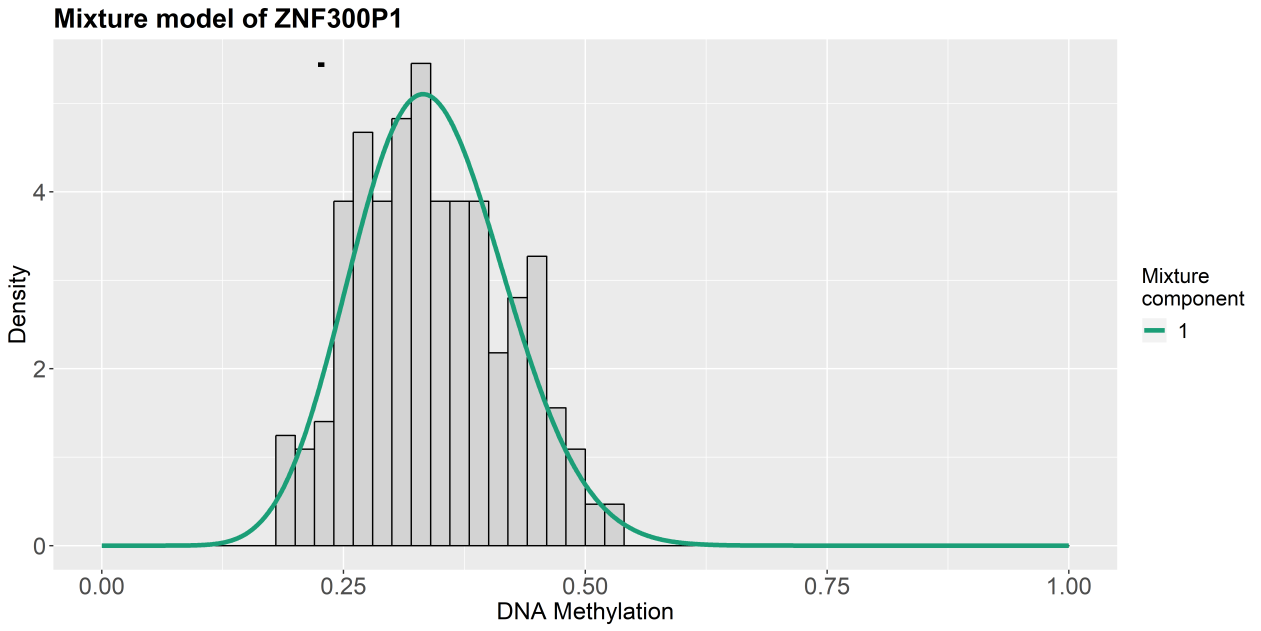

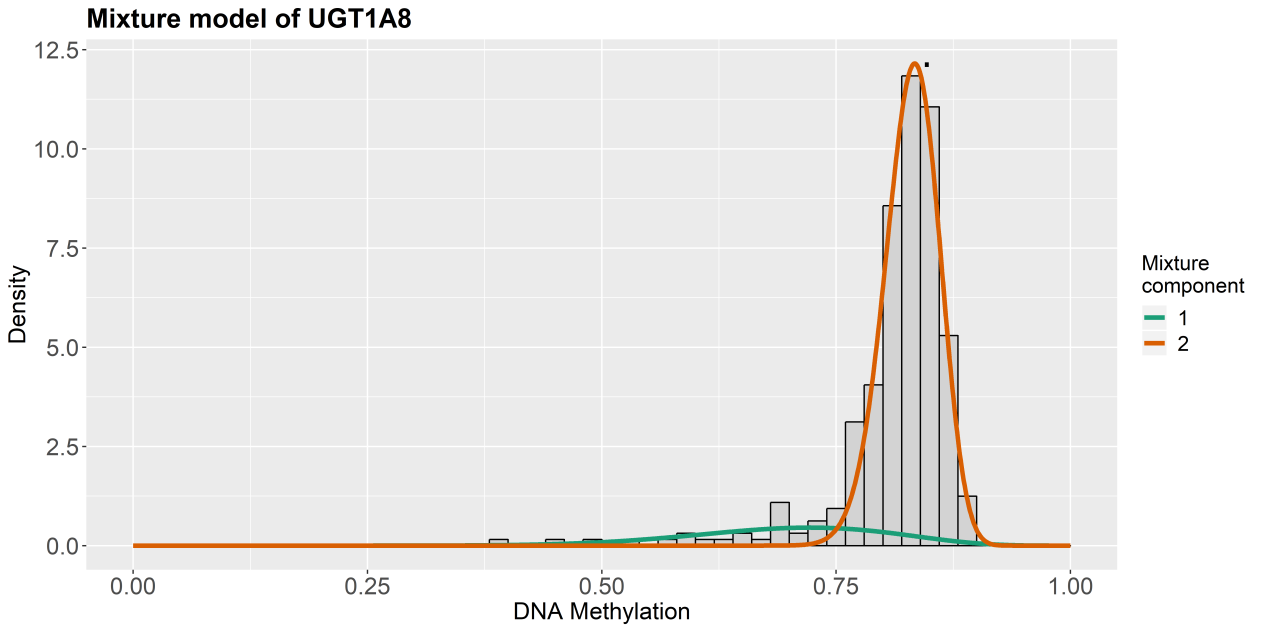


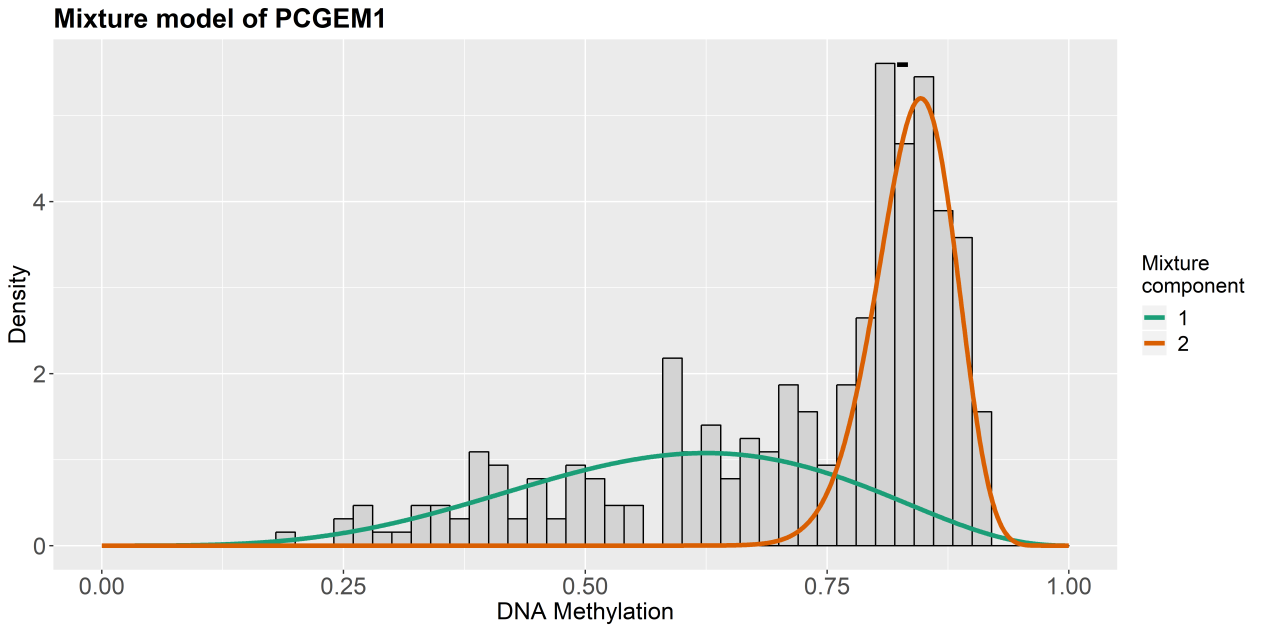

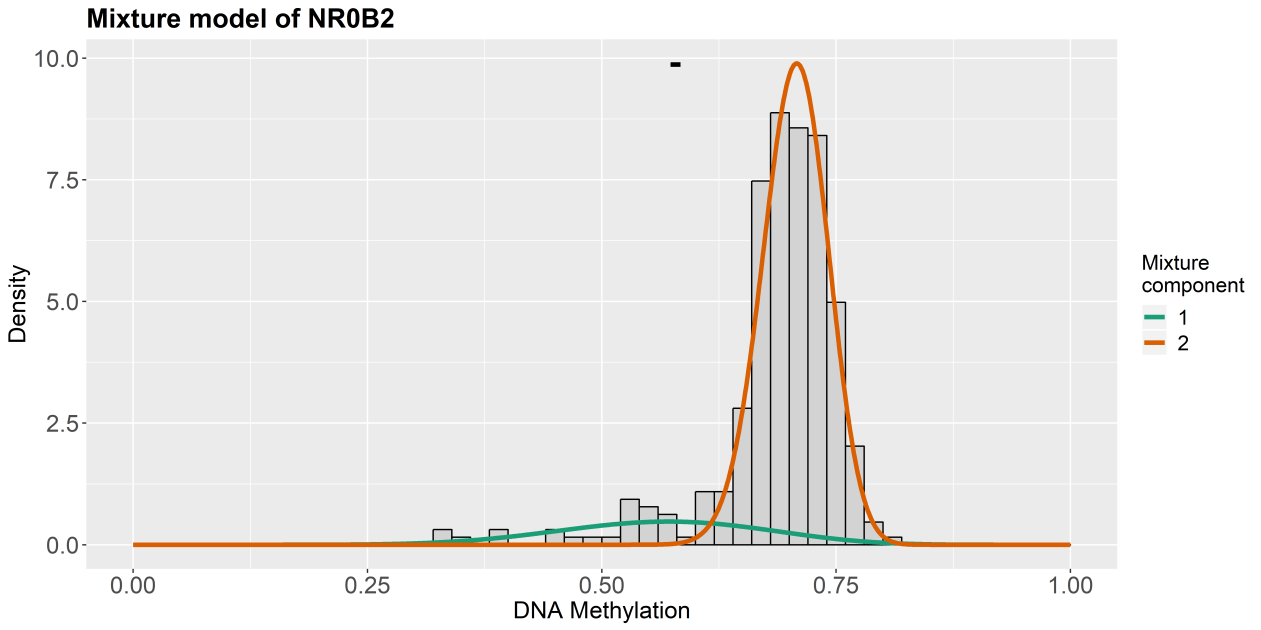

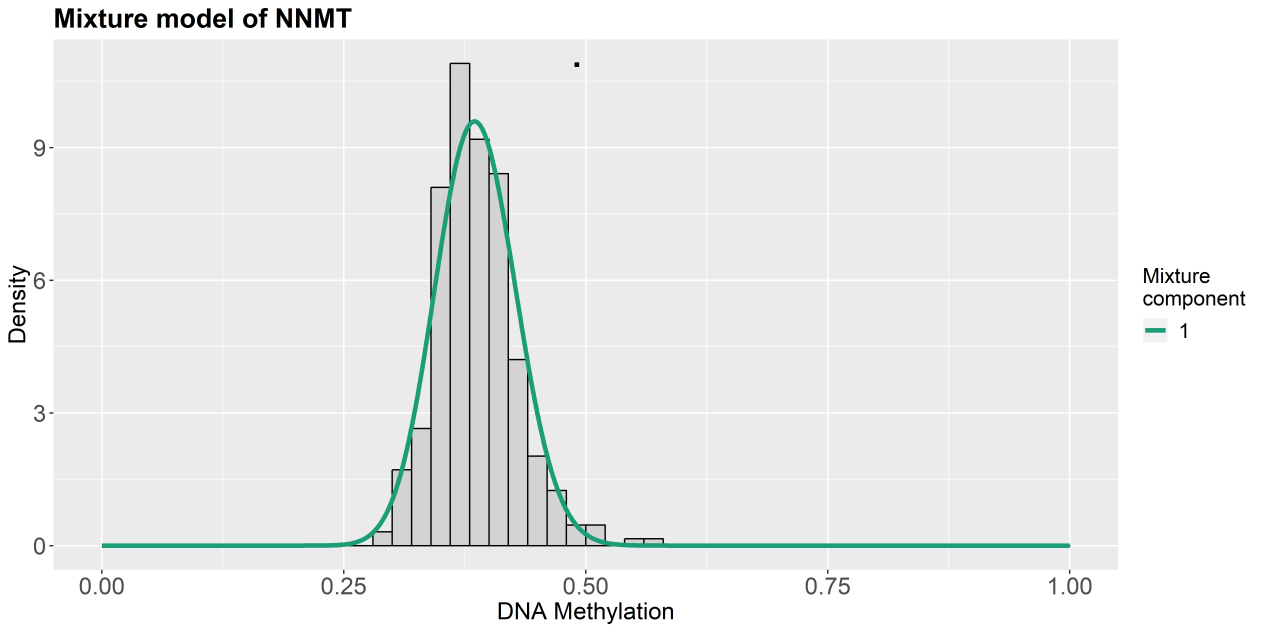

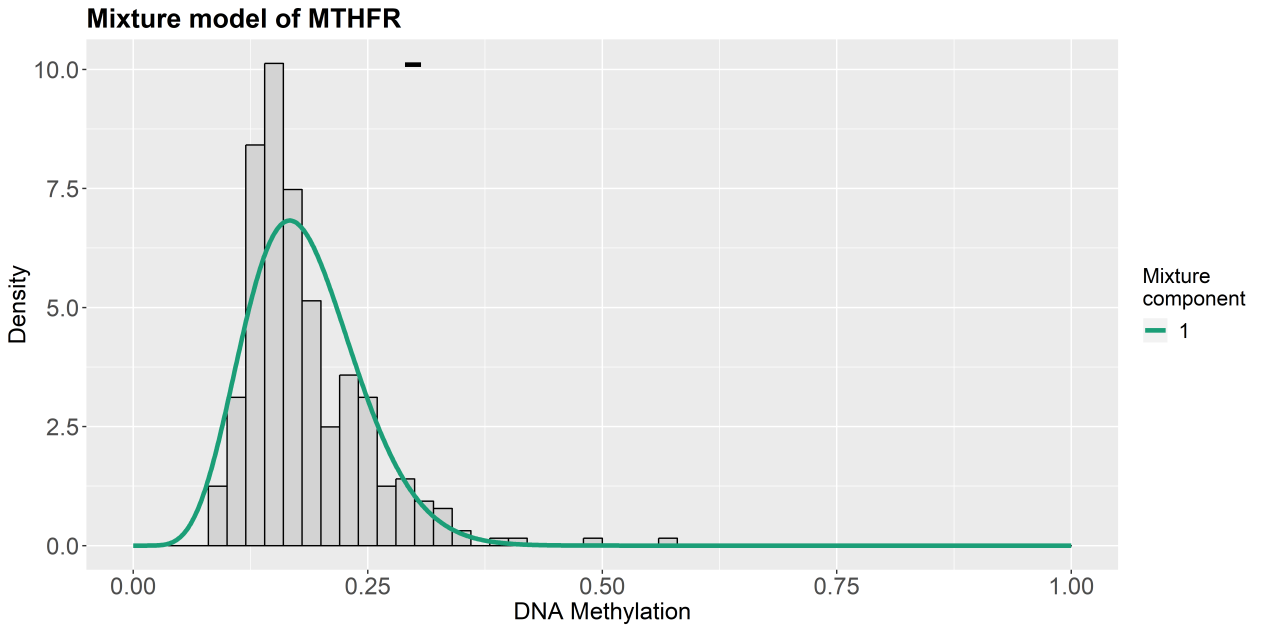

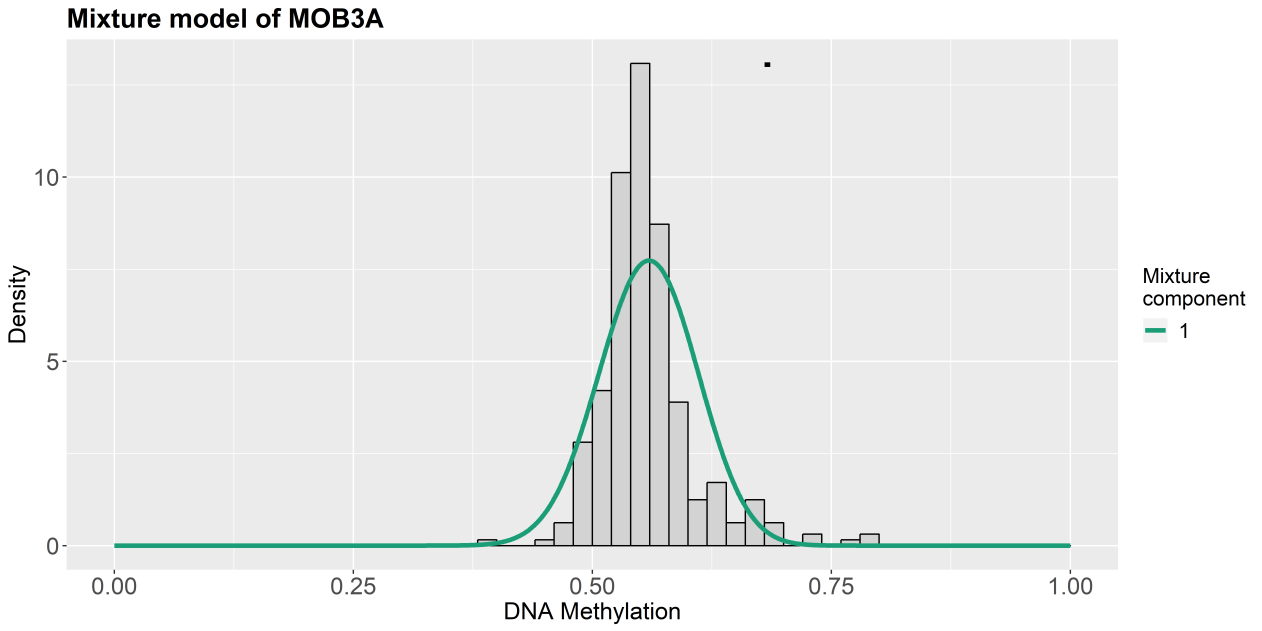

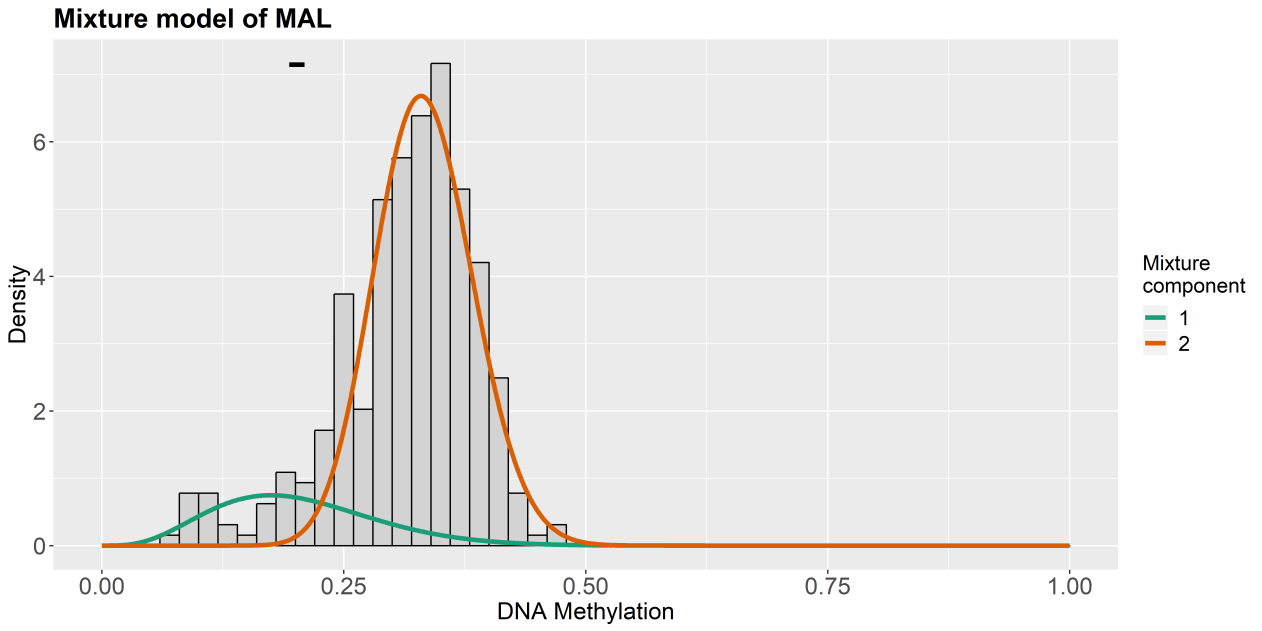

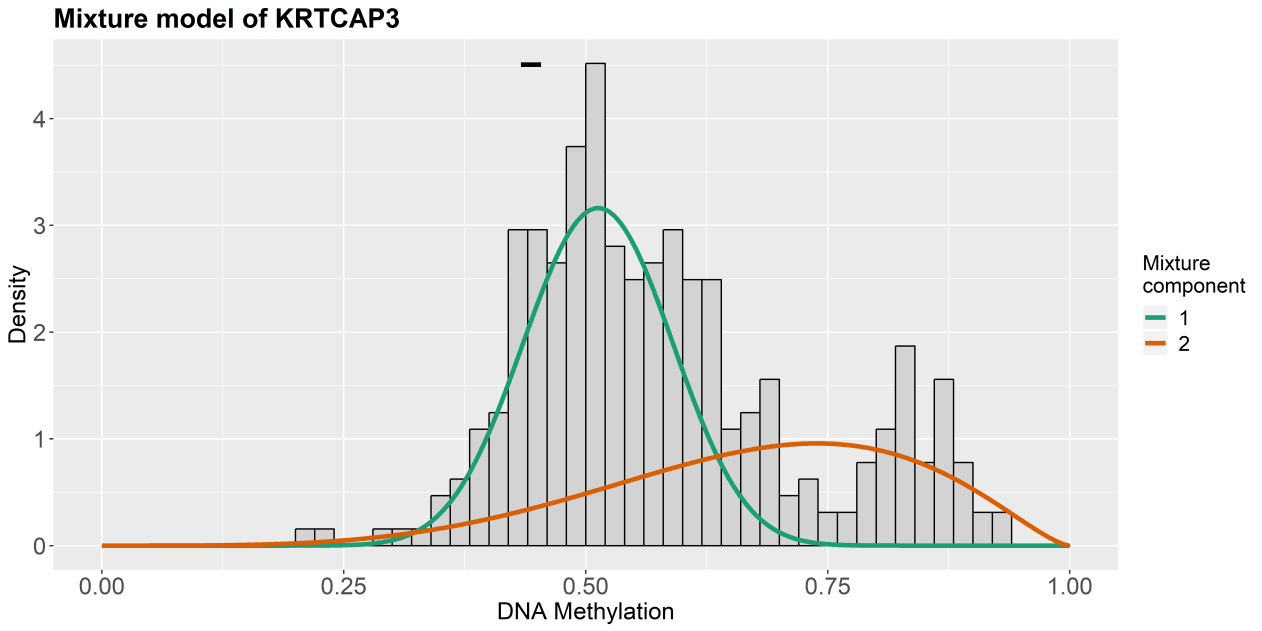

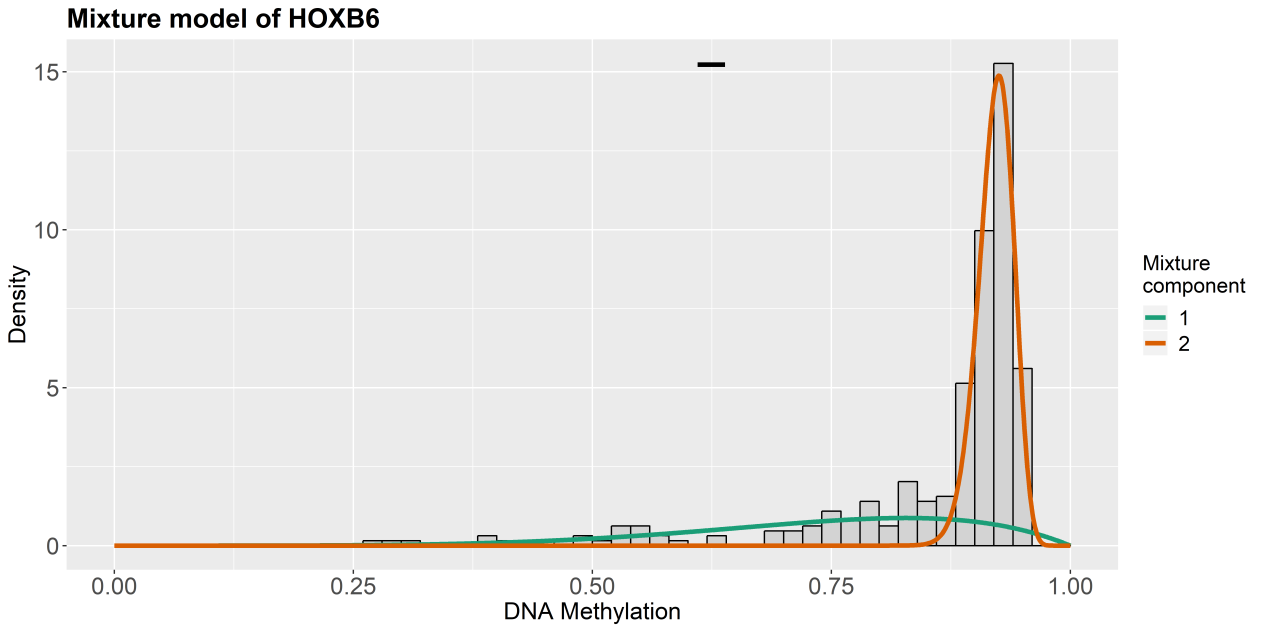

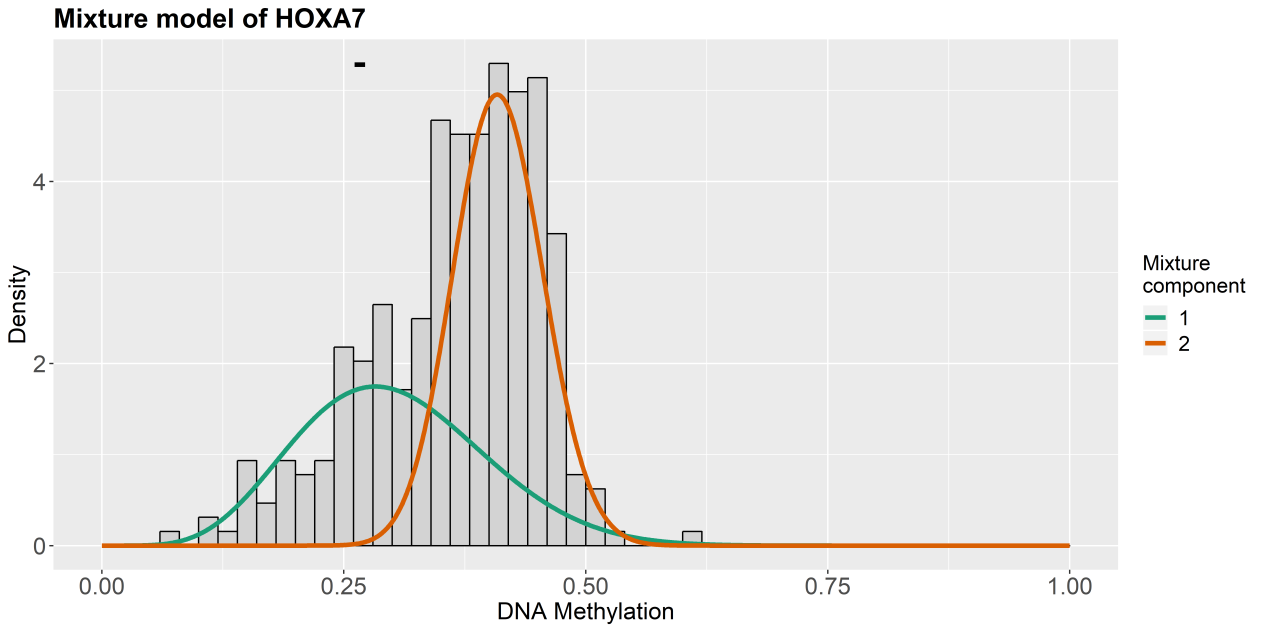

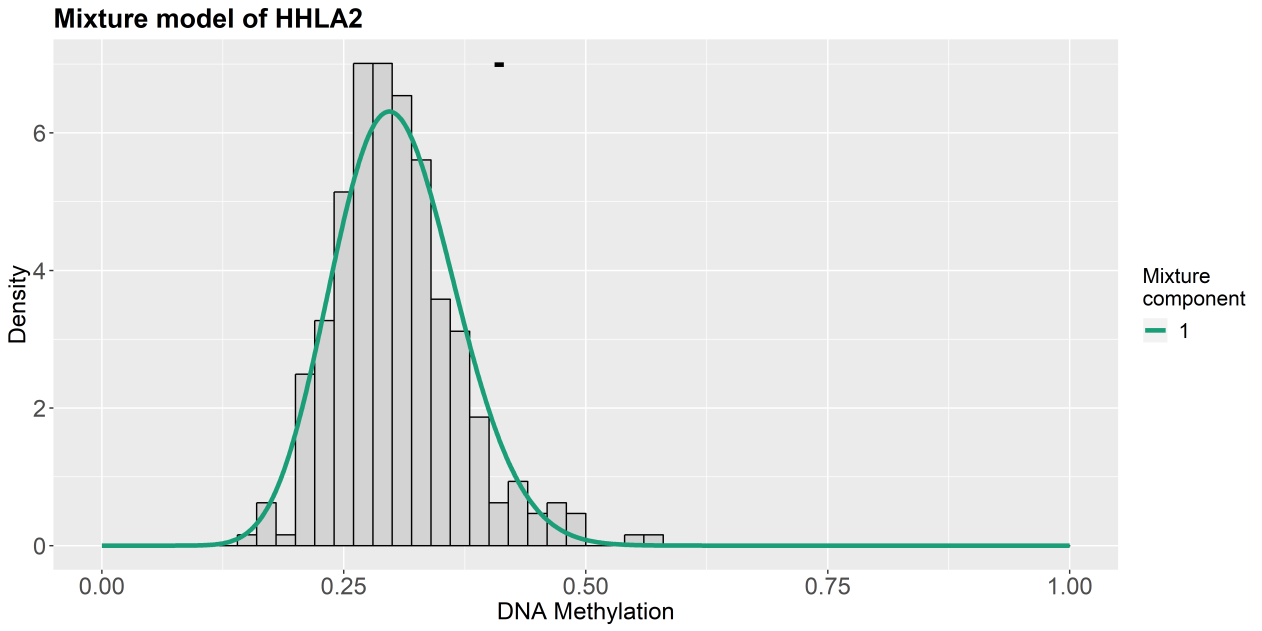

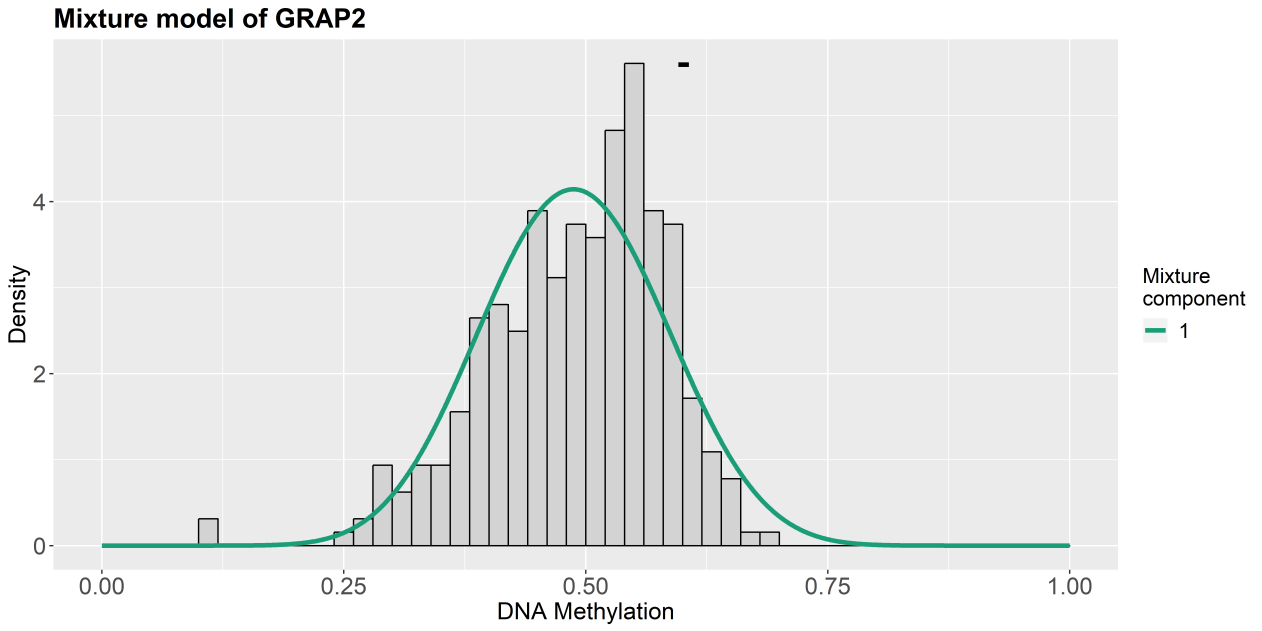

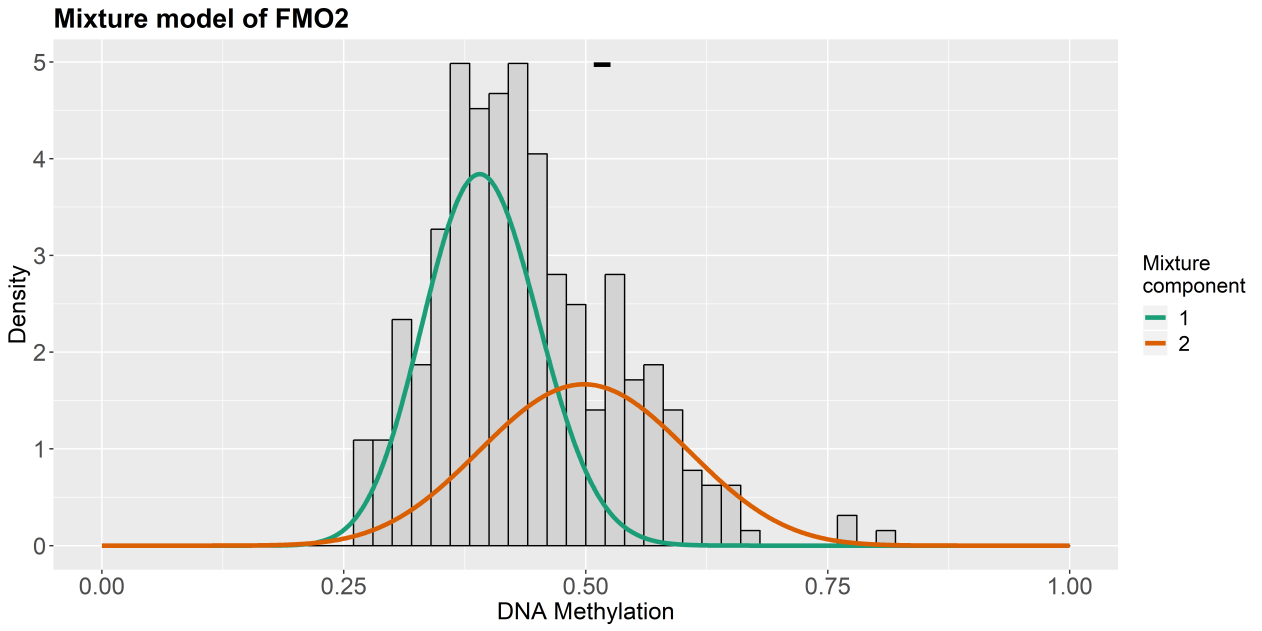

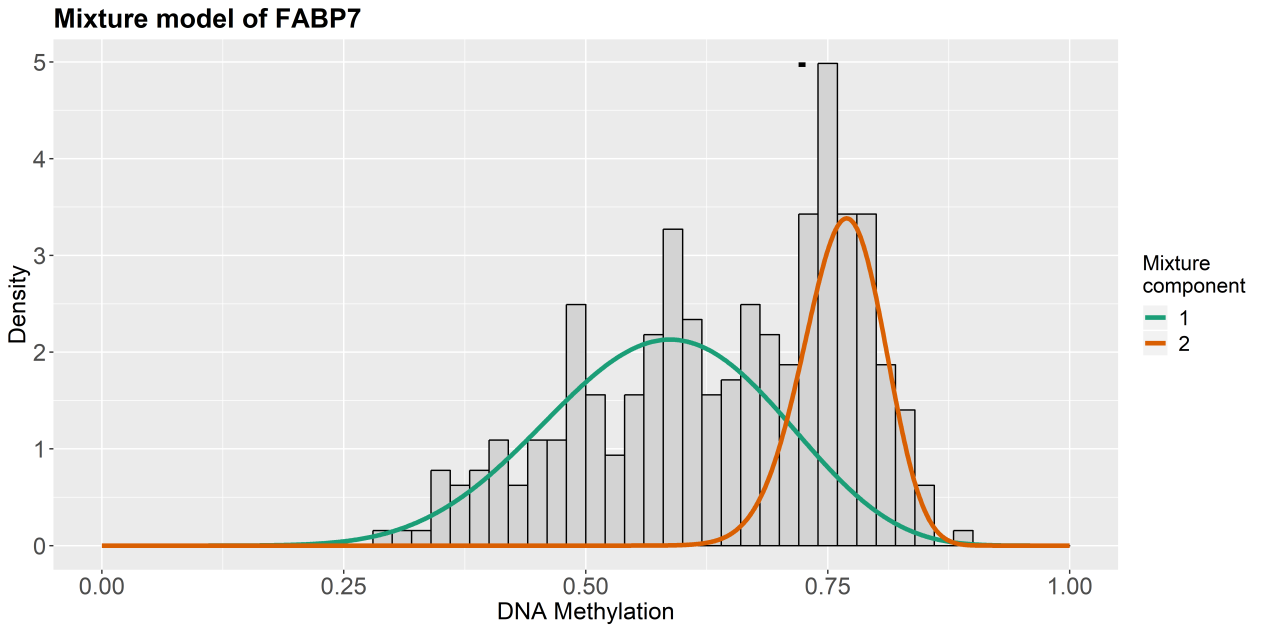

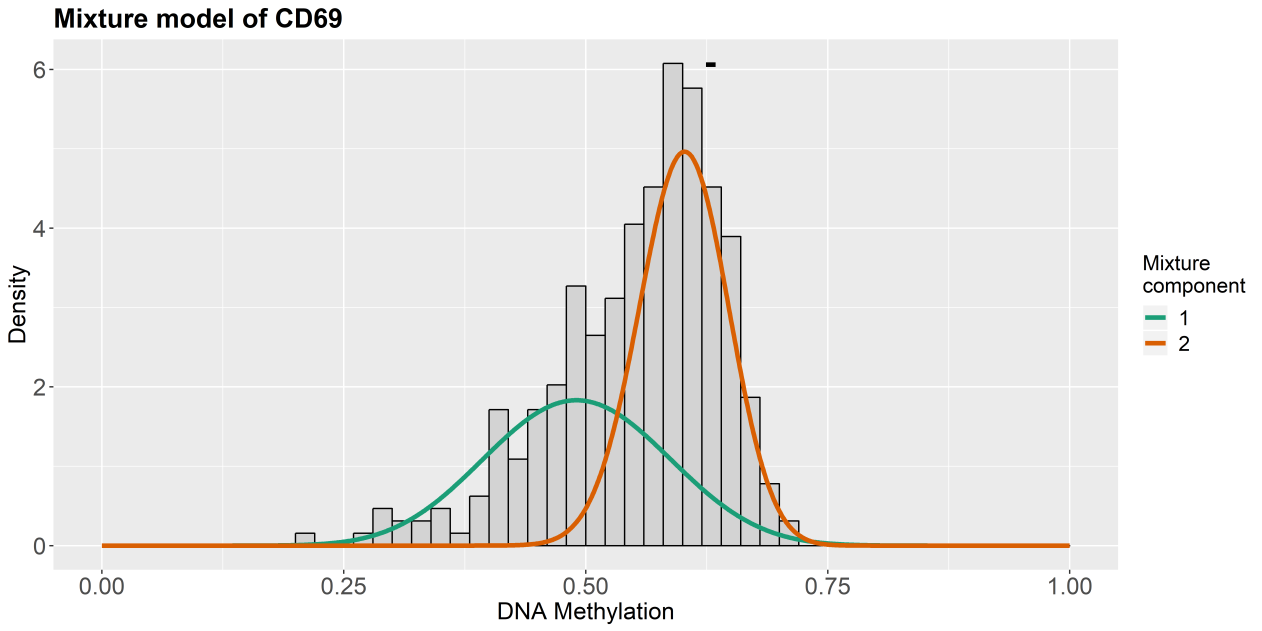

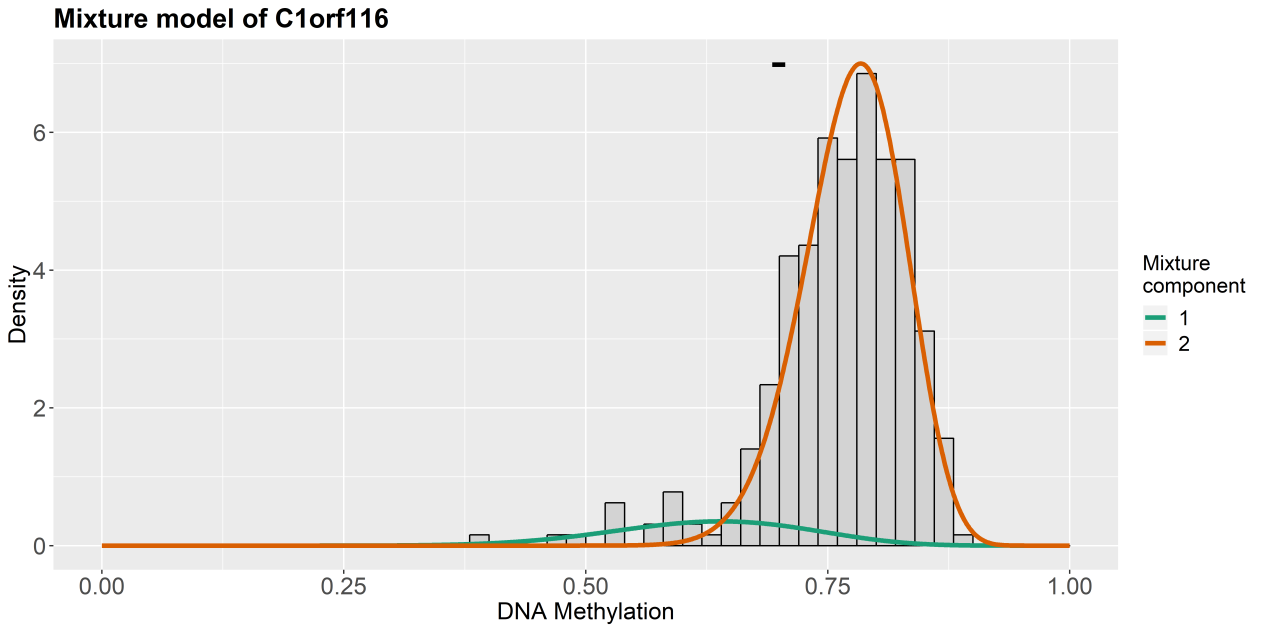

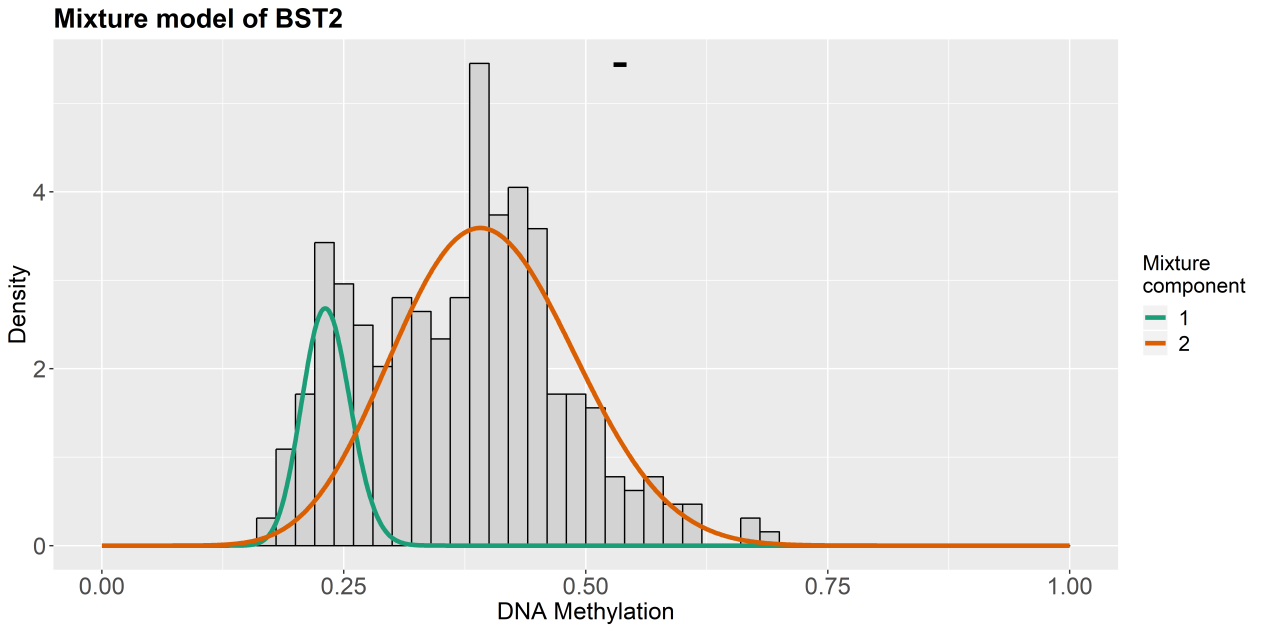

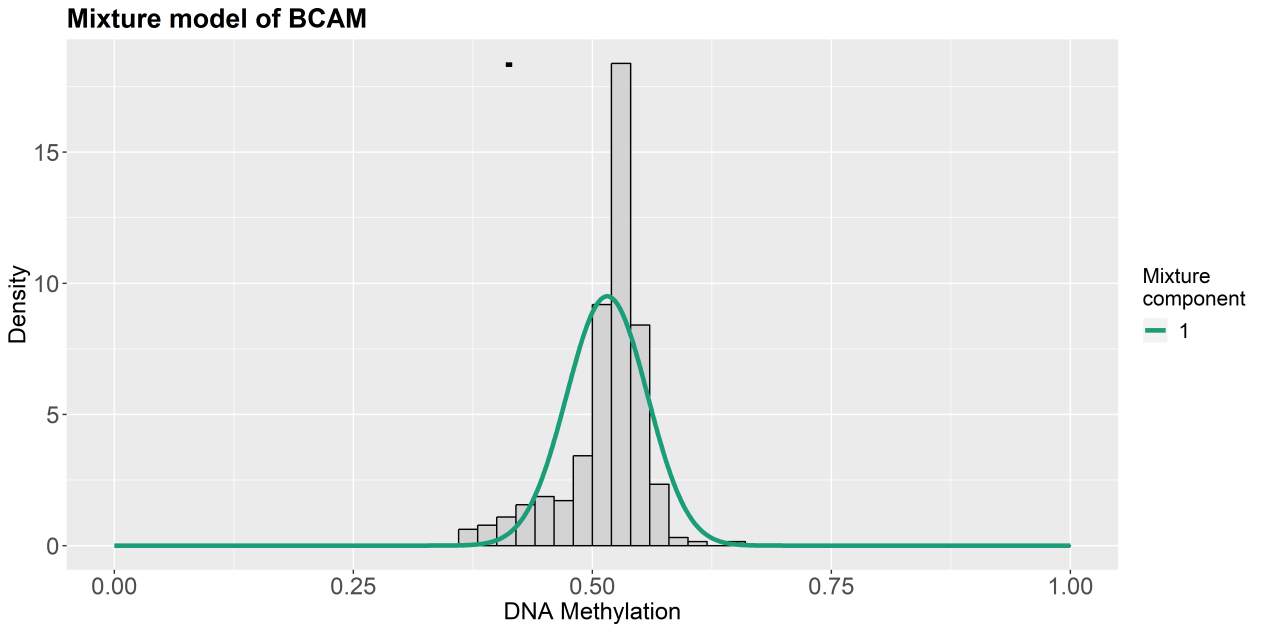

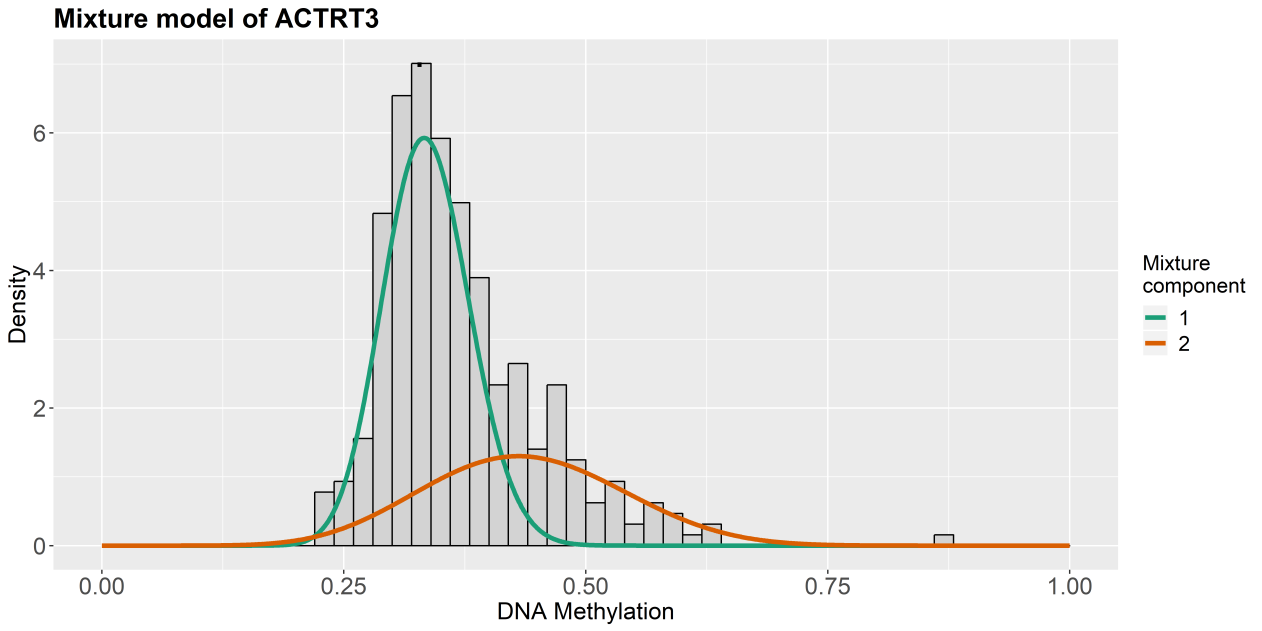

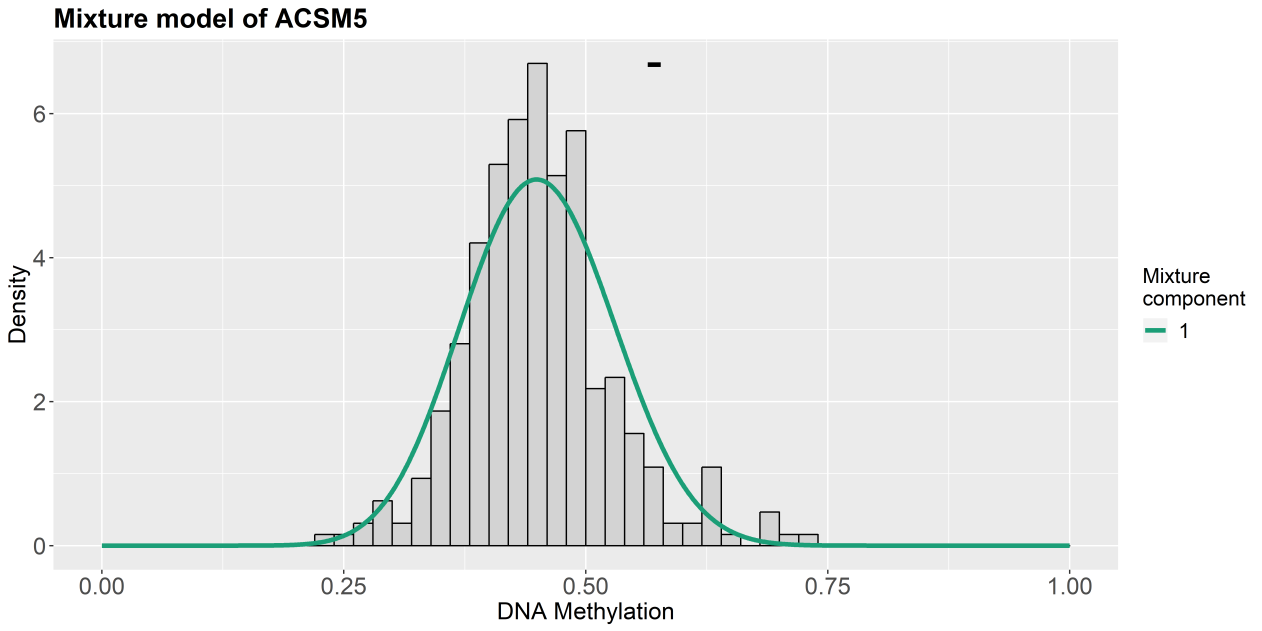

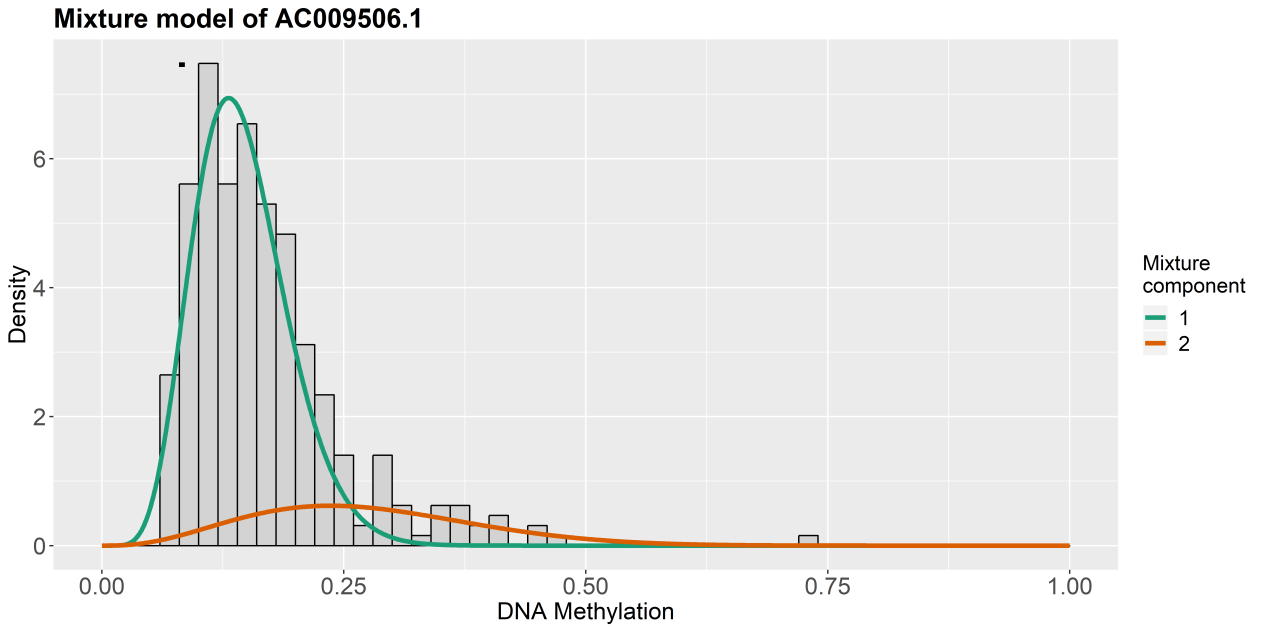

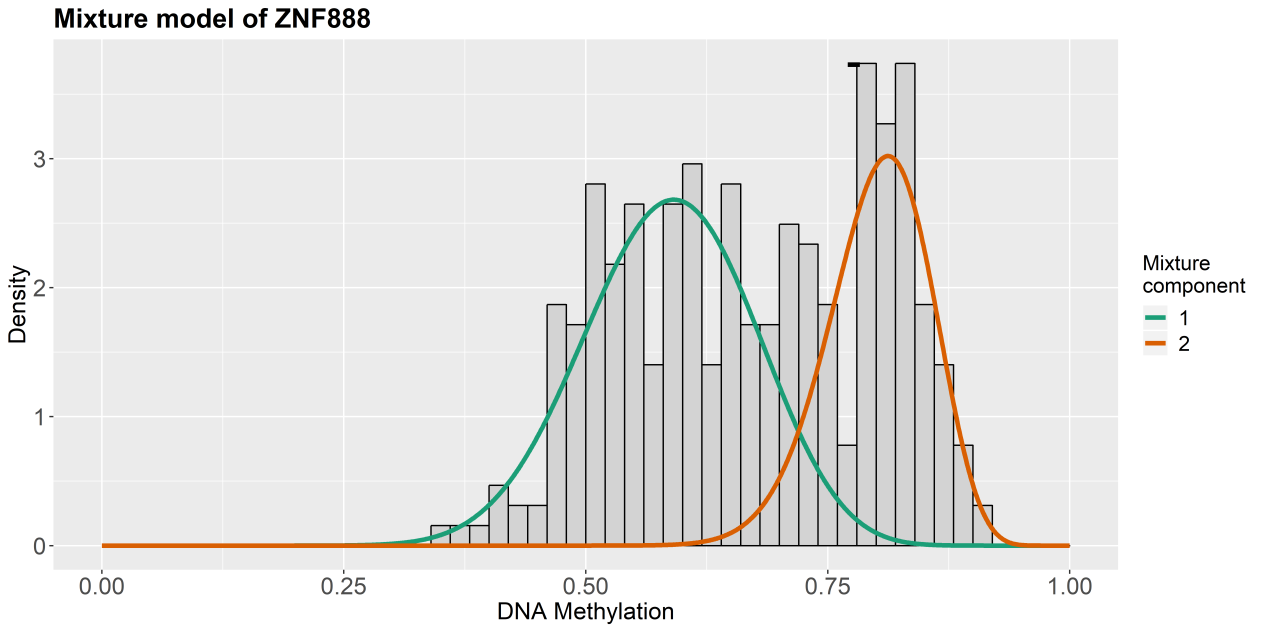

Supplement: Supplementary file 1 — Supporting Information Figure S1: docx. Methyl mixed Model of other methylation‐driven genes. The distribution map represents the methylated status of methylated genes. The histogram demonstrates the distribution of methylation in tumor samples. Horizontal black bars show the distribution of methylation in normal samples [file JCP-235-1296-s001.docx]

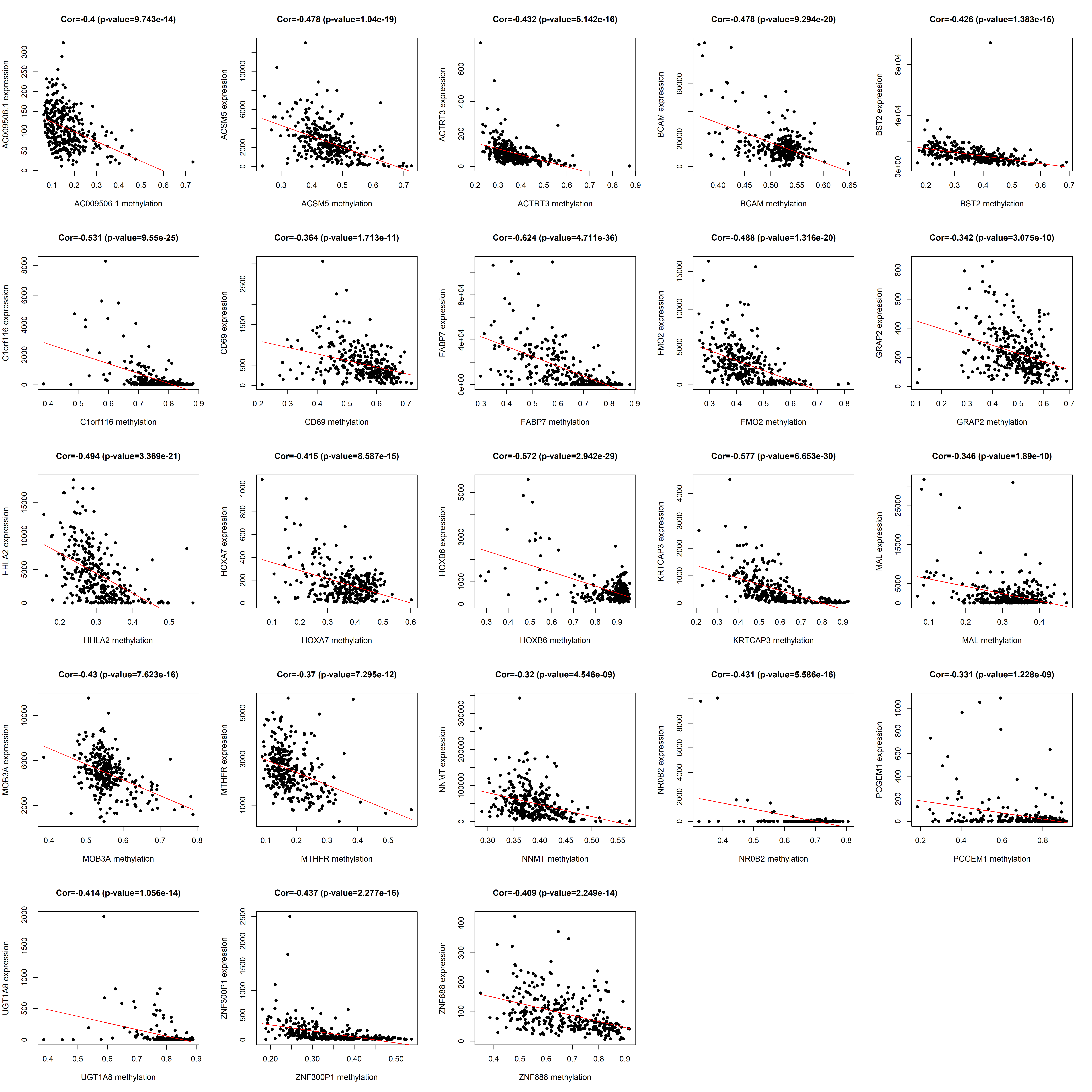

Supplement: Supplementary file 2 — Supporting Information Figure S2: tiff. the correlation between methylation and Gene expression of other genes [file JCP-235-1296-s002.tif]
